# Recognition and Sequencing of Mutagenic DNA Adduct at Single‐Base Resolution Through Unnatural Base Pair

**DOI:** 10.1002/advs.202404622

**Published:** 2024-09-03

**Authors:** Honglei Wang, Wenchao Tie, Wuyuan Zhu, Shuyuan Wang, Ruzhen Zhang, Jianlin Duan, Bingyu Ye, Anlian Zhu, Lingjun Li

**Affiliations:** ^1^ Henan Key Laboratory of Organic Functional Molecule and Drug Innovation Collaborative Innovation Center of Henan Province for Green Manufacturing of Fine Chemicals School of Chemistry and Chemical Engineering Henan Normal University Xinxiang Henan 453007 China; ^2^ State Key Laboratory of Antiviral Drug and Pingyuan Lab Henan Normal University Xinxiang Henan 453007 China; ^3^ Henan Key Laboratory of Organic Functional Molecule and Drug Innovation Collaborative Innovation Center of Henan Province for Green Manufacturing of Fine Chemicals School of Chemistry and Chemical Engineering Key Laboratory of Green Chemical Media and Reactions Ministry of Education Henan Normal University Xinxiang Henan 453007 China

**Keywords:** 3,4‐ethenodeoxycytidine, DNA etheno lesions, sequencing, unnatural base pairs

## Abstract

DNA lesions are linked to cancer, aging, and various diseases. The recognition and sequencing of special DNA lesions are of great interest but highly challenging. In this paper, an unnatural‐base‐pair‐promoting method for sequencing highly mutagenic ethenodeoxycytidine (εC) DNA lesions that occurred frequently is developed. First, a promising unnatural base pair of dεC–dNaM to recognize εC lesions is identified, and then a conversion PCR is developed to site‐precise transfer dεC–dNaM to dTPT3–dNaM for convenient Sanger sequencing. The low sequence dependence of this method and its capacity for the enrichment of dεC in the abundance of as low as 1.6 × 10^–6^ nucleotides is also validated. Importantly, the current method can be smoothly applied for recognition, amplification, enrichment, and sequencing of the real biological samples in which εC lesions are generated in vitro or in vivo, thus offering the first sequencing methodology of εC lesions at single‐base resolution. Owing to its simple operations and no destruction of inherent structures of DNA, the unnatural‐base‐pair strategy may provide a new platform to produce general tools for the sequencing of DNA lesions that are hardly sequenced by traditional strategies.

## Graphical abstract

The 3,4‐ethenodeoxycytidine lesion is highly mutagenic and closely correlated with disease occurrence. The recognition and sequencing are of great interest but remain undemonstrated. Here, the first workflow for recognition, amplification, enrichment, and sequencing of εC lesions via the newly defined εC: NaM unnatural base pair and sequence lesions in real biological samples at single‐base resolution is established.

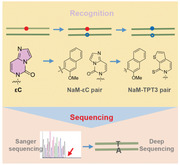

## 1 Introduction

DNA integrity is constantly threatened by various endogenous and exogenous agents such as peroxidation of lipids, environmental pollutants, anticancer drugs, cigarettes, etc.^[^ ^1^ ^]^ The damage events are up to 70 000 in a cell one day, and unrepaired lesions may distort DNA structure, stall replication, or impact gene expression, which are associated with adverse biological consequences, such as cell death and mutation, and are linked to cancer and aging.^[^ ^1e^ ^]^ The recognition and sequencing of special DNA lesions are of great interest in clarifying their roles in different diseases and their potential as biomarkers for cancer risk.^[^ ^2^ ^]^ Current methods for the recognition of lesions in DNA sequence mainly include: 1) to design a chemical or enzymatic reaction to specifically cleave the DNA lesions;^[^ ^2e,f^ ^]^ 2) to utilize the functional groups on the lesions for developing specific bioconjugations;^[^ ^2g,h^ ^]^ 3) to develop specific antibodies that can enrich lesions on the DNA oligos;^[^ ^2i,j^ ^]^ and to utilize new technologies such as nanopore sequencing.^[^ ^3^ ^]^ Through years of effort, the detection and sequencing of some types of DNA lesions such as 8‐oxo‐7,8‐dihydro‐2′‐deoxyguanosine (8‐oxodG) and benzo[a]pyrene dihydrodiol epoxide‐derived dG (BPDE‐dG) have been developed,^[^ ^2e,g,i,j^ ^]^ but other types of lesions remain difficult to be sequenced. This is because lesions with various structure changes in DNA are hard to be recognized. Moreover, it is extremely challenging to differentiate them in complex DNA contexts when the lesions have very similar chemical structures to natural bases, and lack reactive or functional groups.

3,4‐Ethenodeoxycytidine (εC) is such a kind of DNA lesion with similar chemical structures to natural bases and no functional groups. Work published by Bartsch (1981) showed that treatment of single‐strand DNA with vinyl chloride (VC) metabolites could form εC on the cytidine residue.^[^ ^4^ ^]^ Numerous data have shown the accumulations of εC lesions when cells, animals, and human beings were under oxidative stress or exposed to epoxides resulting from the metabolisms of various industrial pollutants.^[^ ^5^ ^]^ εC lesions can cause strong replication blockages and mutagenesis.^[^ ^6^ ^]^ The mutation frequency of εC is unveiled in the yields of 32% in *Escherichia coli* cells and 81% in simian kidney cells.^[^ ^7^ ^]^ They can also cause transcriptional blockages, and the blockages are exacerbated in Cockayne Syndrome and xeroderma pigmentisum patient‐derived lymphoblastoid and fibroblast cells.^[^ ^8^ ^]^ All above suggest a closed relationship between εC lesions and adverse biological consequences and cancer‐prone inflammatory diseases. Despite extensive studies on the physiologic processes of εC lesions, the sequencing method has remained undemonstrated so far.

**Figure 1:**
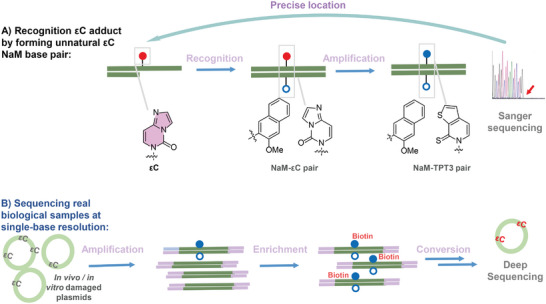
Recognition, amplification, enrichment, and sequencing of εC lesions by the newly defined εC: NaM unnatural base pairs at single‐base resolution.

The field of unnatural base pairs has shown a series of privileged pairs like P–Z, Ds–Pa/Px, 5SICS/TPT3–NaM, etc., that can bioorthogonally replicate, transcript, and translate with high fidelity and efficiency closed to natural base pairs.^[^ ^9^ ^]^ One of the advantages of these unnatural base pairs is that they can be amplified well by PCR, thus offering alien markers on the exact sites in the DNA sequences that can be further sequenced, labeled, or other functionalized. Moreover, unnatural bases can also be used to recognize DNA lesions, such as 3‐methylthymine (m3T) and 2‐hydroxy‐2′‐deoxyadenosine (2‐oxo‐A) lesions.^[^ ^10^ ^]^ Inspired by the merits of unnatural base pairs, we envisaged that the formation of unnatural base pairs with εC lesions bearing desirable efficiency might provide a practical solution for the recognition, amplification, enrichment, and sequencing of this toxic lesion. In this regard, we identified a promising unnatural base pair of dεC–dNaM through primer extension experiments and their kinetic assays. Then, a conversion PCR method was developed to site‐precisely transfer dεC–dNaM to dTPT3–dNaM for convenient sequencing via the Sanger terminal method, which could be further specifically transferred to C–G and A–T pairs enabling deep sequencing. After validating the low sequence dependence of the method and effective enrichment of dεC in the abundance of as low as 1.6 × 10^–6^, we finally demonstrated the successful application of this unnatural‐base‐pair methodology for recognition, amplification, enrichment, and sequencing of εC‐damage biological samples that were generated in vitro and in vivo, respectively (**Figure** **1**). Collectively, this work reports the first single‐base‐resolution sequencing of εC enabled by the dεC–dNaM pair together with its simple and convenient conversion/amplification technology, all these will facilitate the mapping εC lesion for unveiling its roles in multi‐aspect physiological activities with more precise dimension.

## 2 Results and Discussion

### 2.1 Recognition of εC Via Discovering Its Hydrophobic Unnatural Pairing Partners

**Figure 2:**
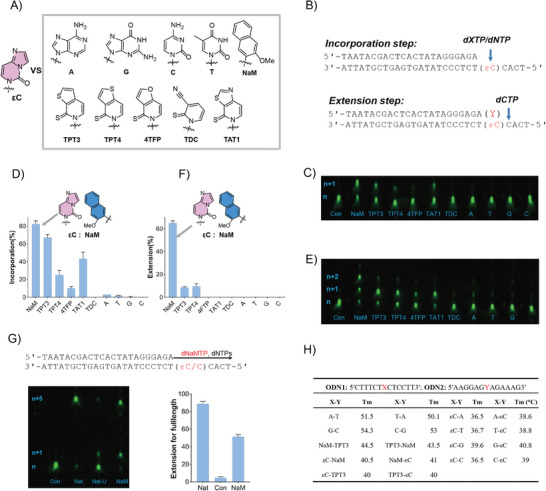
Recognition of εC lesion in DNA oligos with unnatural base nucleotides. A) Structures of natural and unnatural base nucleotides paired with εC, sugar residues are omitted. B) Illustration of single nucleotide incorporation opposite the εC site and primer extension assay. C) Representative gel for incorporations opposite the εC site with different deoxynucleoside triphosphates as shown in (A). D) incorporation level. E,F) Representative gel for the primer extension after incorporations opposite the εC site with different deoxynucleoside triphosphates and extension level. G) Full‐length extension of DNA primer after pairing the εC site with NaM, representative gel and extension levels of full‐length are shown, con: without dNaMTP and dNTPs, Nat: natural C templates with dNTPs, Nat‐U: εC templates with only dNTPs, NaM: εC templates with dNaM and dNTPs. H) Melting temperatures of duplex formation between ODN1 (X) and ODN2 (Y). The data are averages of three independent experiments or mean ± standard deviation (mean ± SD).

We attempted to find a proper molecule that could specifically pair with εC for recognition of them in the DNA sequences. The inherent structure of εC destroys the standard H‐bond form, and thus εC may not pair and mispair with natural bases effectively. Meanwhile, the εC contains a pyridone motif which has also been widely used as the parent structure to develop unnatural base pairs, e.g. P–Z and NaM–TPT3.^[^ ^11^ ^]^ These inspire us to discover pairing partners of εC from a pool of NaM‐type or TPT3‐type compounds listed in **Figure** **2**A.

We performed single‐nucleotide incorporation experiments based on our previous method (Figure 2B–D).^[^ ^12^ ^]^ When all the natural deoxyribonucleoside triphosphates (dNTPs) could be barely incorporated opposite εC with yields of less than 5%, several deoxyribonucleoside triphosphates of artificial bases (dN′TPs) had dramatically increasing yields in such incorporation reactions. dNaMTP had the highest yield for incorporation with 82%. The incorporation yield of dTPT3TP exceeded 60%. For other unnatural bases, the incorporation yields ranged from 10% to 40%, except for TDC with no incorporation. Then steady‐state kinetic experiments were conducted (**Table** **1**; Figure S1, Supporting information). Consistent with the results of single‐nucleotide incorporation experiments, the *V*_max_/*K*_m_ values for incorporations of dATP and dTTP were low as 1.3 × 10^5^ and 4.7 × 10^4^, respectively, and no incorporation bands were observed even though the concentration of the nucleotides was up to 1.0 mM for dCTP and dGTP. In contrast, *V*_max_/*K*_m_ values for incorporations of dNaMTP and dTPT3TP were 2.83 × 10^6^ and 1.85 × 10^6^, 21 and 14 folds higher than dATP. The data demonstrate hydrophobic unnatural bases can be used as effective pairing partners for the εC lesion.

**Table 1:** Steady‐state kinetic assays.

T	dXTP	*V*_max_ (%·min^–1^)	*K*_m_ (µM)	*V*_max_/*K*_m_
εC	NaM	10.51 ± 0.43	3.72 ± 0.58	2.83 × 10^6^
	TPT3	5.07 ± 0.22	2.74 ± 0.62	1.85 × 10^6^
	TAT1	3.59 ± 0.13	4.15 ± 0.65	8.65 × 10^5^
	A	4.51 ± 0.29	33.38 ± 9.61	1.35 × 10^5^
	T	2.08 ± 0.12	44.68 ± 10.27	4.65 × 10^4^
	G	n.d	n.d	n.d
	C	n.d	n.d	n.d

^nd^ represents the kinetic constants that are too low to be detected; T, template; *V*_max_/*K*_m_: %·min^–1^·M^–1^ (*n* = 3, mean ± SD).

Efficient extension ability after recognition of εC by artificial bases is key for obtaining full‐length products. Primer extension assays after incorporation are applied to quantitatively evaluate the extension ability of the formed εC–NaM pair (Figure 2B,E,F). For dTAT1, d4TFP, and all four natural deoxyribonucleotides, no extension bands could be observed even if they could pair with εC to some extent in the incorporation steps. For dTPT3 and dTPT4, only less than 10% of extension bands were observed after pairing with εC. To our delight, the extension rate could exceed 60% after pairing εC with NaM. To test the capacity for obtaining full‐length product, we run the template extension experiments in the presence of four natural dNTPs with or without dNaMTP (Figure 2G). About 50% full‐length products were obtained successfully with dNaMTP and the primer extension was nearly stalled at the εC site without dNaMTP. We also measured the thermal stability to explain the formation of εC–NaM unnatural base pair (Figure 2H; Figure S2, Supporting information). Duplexes containing εC pairing with natural base (A, T, G, C) or unnatural base (NaM and TPT3) were measured with *T*m ranging from 36.5 (εC–C or A) to 41 °C (εC–NaM), in the order of NaM > TPT3 > G > A ≈ T ≈ C, indicating the preference of εC to pair with NaM than others.

### 2.2 Amplification and Sequencing of εC Lesions in Assistance with NaM: TPT3 Pair

**Figure 3:**
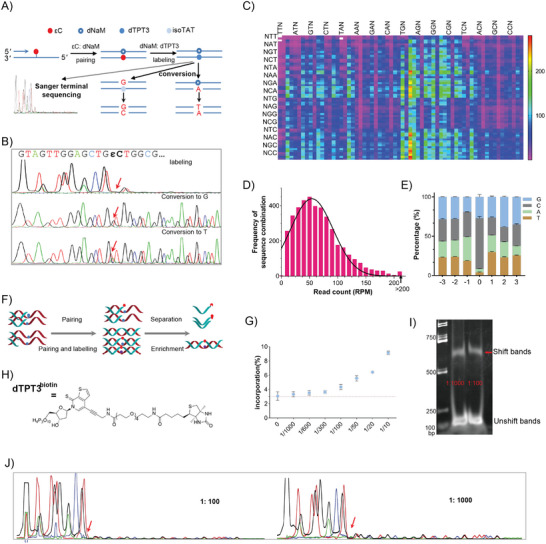
Amplification, sequencing, and enrichment of εC lesions. A) Scheme of locating and sequencing the εC site. B) Sanger sequencing of the labeling and conversion products, only the antisense strands with NaM are shown, the red arrowheads indicate the corresponding εC location with signal termination or conversion to G or T. C) Heat map indicating conversion efficiencies of the bridge base after labeling PCR, three white blocks show the sequence content not detected (TTTεCTTT, GTTεCTTT, and ATTεCTAT), the third bases upstream (left) and downstream (up) of the unnatural base are simplified as “N” and arranged in the order of T, A, G, and C, each sequence context surrounding the εC site is shown, read counts were normalized to reads per 0.25 million (RPM). D) The frequencies of sequence combinations after conversion. E) The distribution frequency of natural bases in each random site and the εC site after conversion. F) Illustration of the enrichment procedure. G) Plot of percentage of single nucleotide incorporation of dNaMTP in a mixture of the εC template and dC template, the red dashed line is background for incorporation into the dC template. H) The structure of dTPT3TP^biotin^. I) Biotin–streptavidin‐based gel mobility assay. The shifted band was marked by a red arrowhead. J) Raw Sanger sequencing data of the second round PCR products after enrichment. Only the antisense strands with NaM are shown, the red arrowheads indicate the corresponding εC location. For (C) and (D), the data present are averages of three independent assays. For (E) and (G), the data present are averages and standard deviations of three independent assays.

We next attempted to build effective amplification and sequencing methods based on the εC–NaM pair. Considering the well‐established sequencing methods of the TPT3–NaM unnatural base pair and its compatibility with commercial Sanger sequencing instruments,^[^ ^11b^ ^]^ the strategy to transfer εC–NaM pair to TPT3–NaM and then utilize the sequencing methods of TPT3–NaM should be most economic at the current stage. The Sanger‐terminal sequencing method of TPT3–NaM was used to identify the precise location of εC adduct in a synthetic 63‐mer sequence (**Figure** **3**A; Table S1, Supporting information). After PCR amplification, Sanger sequencing data showed the sharp terminal signal at the exact site of εC (Figure 3B; Figure S3, Supporting information), indicating the effectiveness and accuracy of this method. The PCR efficiency for the transfer of εC–NaM was also analyzed. We showed that the amount of template could be as low as 0.1 pg (Figure S4, Supporting information). On the other hand, the Sanger‐terminal sequencing method is not appropriate to directly sequence multiple TPT3–NaM pairs in DNA oligos, for which we have recently developed a bridge‐base strategy to transfer TPT3–NaM pair to C–G or A–T.^[^ ^13^ ^]^ In the bridge‐base strategy, isoTAT can pair with NaM, as well as G during the replication. So, in the presence of disoTATTP, the TPT3–NaM pair in the templates will site‐specifically convert to C–G pair in the amplicon. Besides, taking advantage of the preference of the NaM base itself, the TPT3–NaM pair can be converted to A–T in the amplicon in the absence of dTPT3TP. With the assistance of such two condition‐dependent conversions, we can realize the sequencing of multiple NaM–TPT3 unnatural base pairs at unknown sites of DNA. Herein, we repurposed this strategy to the sequencing of εC. We transferred εC–NaM pair to TPT3–NaM pair and then TPT3–NaM pair to different nature signals via two‐round PCR (Figure 3A). Sanger sequencing data of PCR products showed signals of C–G or A–T at the locations of TPT3–NaM, respectively (Figure 3B; Figure S3, Supporting information). Therefore, the bridge‐base strategy is also appropriate to sequence εC marked by TPT3–NaM, which means the current method can also sequence multiple εC lesions in a DNA context.

### 2.3 Sequence‐Dependence Analysis of εC–NaM Unnatural Base Pairing

DNA lesions may occur in any complex DNA context and have shown some sequence preferences for both their occurrence and repair.^[^ ^2c^ ^]^ To this end, we synthesized a library bearing NNNεCNNN to investigate the possible sequence dependence for the insert of NaM opposite εC and following extension processes in order to further evaluate the sequence‐dependence property of our sequencing method. PCR with the library was run as above and transferred to G–C was implemented and sent for deep sequencing. Briefly, the data demonstrated that NaM–TPT3 could label 4093 of all the 4096 sequence content of the oligos bearing εC (TTTεCTTT, GTTεCTTT, and ATTεCTAT are the sequence content not detected), which guaranteed the generality of our method for sequencing εC on almost all sequences. The heat map indicated the conversion efficiencies of each possible sequence (Figure 3C), and the frequencies of sequence combinations showed a rational closed‐to‐bell‐shaped frequency distribution around the medium number (61) (Figure 3D). These indicate that the εC–NaM pair is of low sequence dependence. In addition, the Single Nucleotide Polymorphism of three random sites downstream (+) and upstream (–) of the εC site and the detailed dinucleotide sequence distribution also showed some sequence bias (Figure 3E; Figure S5 and Table S2, Supporting information). All these will provide useful references for the deep sequencing of biological samples.

### 2.4 Enrichment of εC Lesions by Biotin–Streptavidin Assay

Low abundance is another obstacle to sequencing εC lesions in real biological samples. We used a biotin‐labeled TPT3 (dTPT3TP^biotin^) as the additional tool to implement an enrichment procedure (Figure 3F,H). First, to define if εC could be recognized at low amounts and ratios on the DNA, a primer extension reaction with various dilutions of the εC template and a constant concentration of dC template was carried out (Figure 3G; Figure S6, Supporting information). We found that the extended products were increased along with the increased εCtemplates, even when the smallest amounts of the εC template (0.03 pmol) were mixed. This result indicates that NaM can be specifically paired with εC in low abundance of the εC template. Second, dTPT3TP^biotin^ and dNaMTP were used to recognize and mark the εC site on the DNA template through PCR amplification. DNA templates were a mixture including DNA containing εC site, natural DNA, and excess genomic DNA fragments, making the εC at an abundance level of 1:100 or 1:1000. After PCR amplification, the εC‐containing products were proven by biotin–streptavidin‐based strand shift assay using nondenatured PAGE (Figure 3I), and then products were enriched by streptavidin magnetic beads; finally, the enriched products were sent for a second amplification and sequenced. We found that the εC at low abundance could be effectively enriched and then transferred to natural bases (Figure 3J; Figure S7, Supporting Information). This effective enrichment makes another important step toward sequencing εC in real biological samples.

### 2.5 Sequencing εC Lesions in the In Vitro and In Vivo Damaged DNA Samples

**Figure 4:**
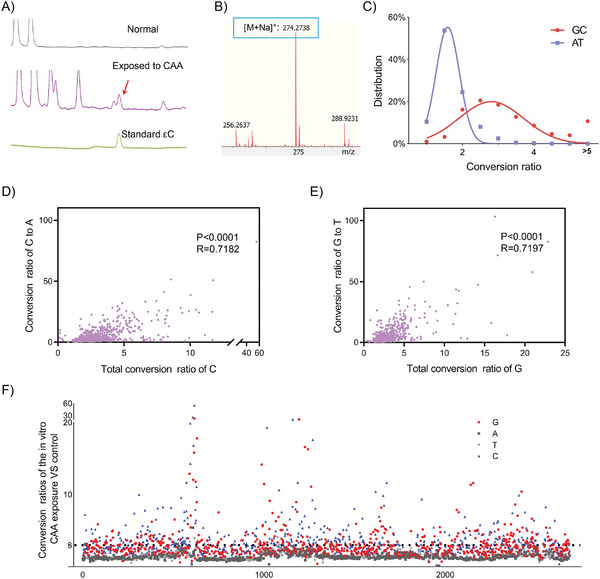
Sequencing of εC lesions in the pUC‐19 plasmid directly exposed to CAA. Detection of the εC lesions using A) HPLC and B) MS. The red arrowhead indicates the εC lesion nucleosides. The peak of εC nucleosides only appears from plasmids exposed to CAA. C) The distributions of GC and AT conversion ratios. Correlation analysis between the conversion ratio of D) C to A or E) G to T and total conversion (C to A: 0.7182, *P* < 0.0001; G to T: *R* = 0.7197, *P* < 0.0001). F) Distribution of the εC lesion sites, the black dashed line is value 3. For (D), (E), and (F), the data present are averages of three independent assays.

We used the in vitro damaged DNA to test the effectiveness of our εC–NaM pair‐based method. pUC‐19 plasmids were directly exposed to chloroacetaldehyde (CAA) to induce the εC lesions.^[^ ^14^ ^]^ The occurrence of εC lesions in the plasmid was monitored by high‐performance liquid chromatography (HPLC) and identified by mass spectrum (MS) after enzymatic digestion (**Figure** **4**A,B). To investigate the distribution of εC lesions, ten pairs of primers were designed to cover the whole plasmid (Figure S8, Supporting Information). Labeling and enriching steps were performed as described above, and the conversion PCR amplification was performed to convert the lesion site to A and its complementary partner G to T for deep sequencing^[^ ^13^ ^]^ (Figure 3B; Figure S3, Supporting Information). To demonstrate the degree of εC's occurrence, the conversion ratio defined by the ratio value of the mutation on the same site of damaged samples to that of undamaged samples was calculated. Both the AT and GC conversion ratios showed standard bell‐shaped frequency distribution (Figure 4C), and the conversion ratios of GC were of dramatically higher level, which suggested the occurrence of εC lesions with high frequency. The total conversion ratios of G or C were highly correlated with conversion ratios of G to T or C to A with coefficients *R* = 0.7197 or 0.7182 (*P* < 0.0001) (Figure 4D,E), respectively, indicating that εC lesion was successfully marked and converted. The distribution of conversion ratios for each base site was shown in Figure 4F, in which the conversion ratios >3 were set down as lesion sites. Based on the data, 558 εC lesions were identified among all the 1364 C sites (Figure 4F; Table S3, Supporting Information). We have reported an analysis method for unveiling the DNA base damaged regions with high occurrence abundance through reading out the signal attenuation from the data of Sanger sequencing.^[^ ^10a^ ^]^ Here, the method is also used to recognize the εC damaged regions to further verify our sequencing method. We designed another four pairs of primers that covered the entire plasmid (Figure S8, Supporting Information), and then amplified, detected by biotin–streptavidin‐based strand shift assay (Figure S9, Supporting Information), enriched by streptavidin magnetic beads, and sequenced them. The damaged sequence would contain a mixture of DNA with εC at different sites (Figure S10A, Supporting Information). So signal attenuations in real samples may show mild and sudden changes due to the distribution of εC lesions with variant abundances (Figure S10B, Supporting Information), in which the signal terminals may not appear as shown in the model, but can be used to recognize DNA base damaged regions with high abundance. To our delight, the regions of detectable signal attenuation matched well with the regions containing εC lesion sites from our current method in Figure 4F; Figure S11, Table S4, Supporting Information. The results demonstrate the current method is reliable for sequencing εC lesions and offers an advanced version of the UBPs‐based lesion sequencing method which updates the sequencing resolution to a single‐base level.

**Figure 5:**
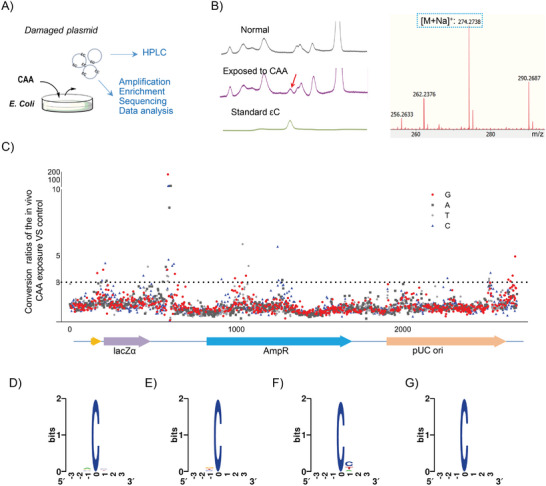
Sequencing of εC lesions in the pUC‐19 plasmid extracted from *E. coil* exposed to CAA. A) The workflow for harvesting damaged plasmid. B) Detection of the εC lesions by HPLC and MS. The red arrowhead indicates εC lesion nucleosides. The peak of εC nucleosides only appears from plasmids extracted from *E. coil* exposed to CAA. C) Distribution of the εC lesion sites, the black dashed line is value 3, the data present are averages of three independent assays. D) Sequence logo of εC lesion sites in plasmids exposed to CAA. E) Sequence logo of C sites without damage in plasmids exposed to CAA. (F) Sequence logo of εC lesion sites in plasmids from *E. coli* exposed to CAA. G) Sequence logo of C sites without damage in plasmids from *E. coli* exposed to CAA. DNAlogo is drawing by http://weblogo.berkeley.edu/logo.cgi.

Finally, we applied our method to sequence the in vivo damaged DNA as the real biological samples (**Figure** **5**A). pUC‐19 plasmids were extracted from CAA‐exposed *E. coli* DH5α cells,^[^ ^15^ ^]^ and the emergence of εC lesion was also monitored by HPLC and identified by MS after enzymatic digestion (Figure 5B). The steps for recognition, amplification, enrichment, and deep sequencing were performed similarly as shown above. Intriguingly, 29 of the 1364 C sites were identified as the εC lesions (Figure 5C; Table S5, Supporting Information) with conversion ratios >3. The consistency with the signal‐attenuation method was also demonstrated, the strong signal attenuation regions were matched with the lesion sites identified from this method (Figure 5D; Figures S12 and S13, Table S4, Supporting Information). The fragments from primer 3 held signal attenuation at some sites bearing the conversion ratios of 2.5–3, which might come from the loss of some sites with lower damaged levels, and there might be a few lesion sites not detected. So, the current sequencing method could also be used to reflect the degree of εC lesions to some extent, except to give the exact occurrence sites. Besides, we also compared the distribution of εC lesions that were induced by in vitro and in vivo methods respectively. There was hardly any sequence preference in the in vitro damaged DNA fragments, but the sequence with another C downstream shown preferably in the occurrence of εC lesions in the in vivo damaged DNA fragments (Figure 5E–H; Figure S14, Supporting Information). The distribution of εC lesions was comparable with similar patterns in some regions and was different in the other regions, and was also clustered in some regions, e.g., a region mainly downstream of the pUC ori showed similar damaged characteristics (Figure S14, Supporting Information), indicating that some regions might be hotspots for the occurrences of εC lesions and the significance consequence for mapping the lesions in living organisms.

## 3 Conclusion

In summary, our work establishes the first practical workflow of recognition and sequencing εC lesions (Figure S15). The merits of this method include high sensitivity (sequencing both the model samples and real biological samples with εC at low abundance), no destruction of the inherent constructures of DNA lesions and natural nucleobases, full compatibility with the readily accessible Sanger sequencing and deep sequencing technology, only PCR and streptavidin‐based enrichment assays with no specialized instruments, low sequence dependence, etc. Therefore, it will provide a general method to get the landscape of εC in genes and facilitate understanding the influence of the εC lesion sites on their biological functions in different research fields.

## 4 Experimental Section

### General Remarks

The phosphoramidite building blocks for εdC, dNaM, and dTPT3 were synthesized according to the method described in the literature.^[^ ^16^ ^]^ Oligonucleotides with modified phosphoramidites were synthesized and sequenced by Sangon Biotech. dNaMTP, dTPT3TP, dTPT3TP^biotin^, and other chemical compounds used were synthesized as reported.^[^ ^11b^ ^] 1^H, ^13^C, and ^31^P NMR spectra were performed on AVANCENanoBay (400 MHz) and Bruker AVANCE III HD (600 MHz). HRMS were performed on Bruker compact Ultrahigh resolution electro‐spray time‐of‐flight mass spectrometry. dNTPs, DNA gel extraction kit, RNase‐free water, and Streptavidin were purchased from Solarbio. Klenow fragment DNA polymerase І was purchased from ABclonal Technology Co., Ltd. OneTaq 2 × Master Mix, OneTaq DNA polymerase, Deepvent DNA polymerase, and Nucleoside Digestion Mix were purchased from New England Biolabs. *E. coli* DH5α cell and pUC‐9 plasmid were purchased from Beijing Zoman Biotechnology Co., Ltd. YeaRed Nucleic Acid Gel Stain was purchased from Yeasen Biotechnology Co., Ltd (Shanghai). Dynabeads MyOne Streptavidin C1 was purchased from Thermo Fisher Scientific.

### Pre‐Steady‐State Kinetic Analysis of Single Nucleotide Incorporation

The pre‐steady‐state kinetic analysis of single nucleotide incorporation was conducted based on literature methods.^[^ ^10a^ ^]^ The 23‐mer primer labeled with HEX and 28‐mer DNA template with ɛC lesion in the 24th position were shown in Table S1. The primer and template were annealed by incubating at 95 °C for 3 min, 48 °C for 20 min, and then 25 °C for 10 min. A total of 2.25 pmol template‐primer, 4.5 U Klenow fragment of *E. coli* DNA Polymerase I (Kf (exo^–^)) were added to the 1 × reaction buffer (50 mM NaCl, 10 mM Tris‐HCl, 10 mM MgCl_2_, 1 mM DTT, pH 7.9 at 25 °C) in a final volume of 20 µL, the mix was preheated at 37 °C for 1 min. Subsequently, 3 × dNTP/dXTP (9 µM) in the 1× reaction buffer was added to initiate the reaction. The reaction was maintained at 37 °C for 15 s, then 10 µL 0.05 M EDTA (pH 8.0) was added to quench the reaction at 80 °C for 3 min. To define if ɛC could be specifically recognized under low abundance conditions, an additional assay was carried out with a constant concentration of dC template and various dilutions of the εC template (30 pmol dC: 0.03, 0.05, 0.1, 0.3, 0.6, 1.5, 3 pmol ɛC). The reaction system was evaporated under reduced pressure at 45 °C, and residual solids were dissolved again with 4 µL 1 × loading buffer (90% formamide, sufficient amounts of bromophenol blue, and xylene cyanol), detected on 15% denaturing urea polyacrylamide gel electrophoresis. The gel was imaged on Amersham Imager 680, and the primer (called the *n* bands) and extended primer (called the *n* + 1 bands) fluorescence intensities were quantified by Amersham Imager 600 Analysis Software. The incorporation efficiency was calculated as a percentage of the *n* + 1 band. All the pre‐steady‐state kinetic analyses were independently repeated three times, the data presented are averages and standard deviations of three independent assays.

### Steady‐State Kinetics Assay of Single Nucleotide Incorporation

The single nucleotide incorporation was performed under steady‐state conditions with changes, including DNA polymerase concentration, primer‐template concentration, and reaction time, based on the literature.^[^ ^17^ ^]^ Briefly, the HEX‐labeled primer and DNA template were annealed following the method in pre‐steady‐state kinetic analysis. Then, 9 pmol primer‐template complex and 0.45 U DNA polymerase were added to 1 × reaction buffer in a total volume of 20 µL. The reaction system was incubated at 37 °C for 1 min and was immediately added gradient concentrations of nucleotides to initiate the reaction, respectively. The details of nucleotides concentrations were as follows: dNaM, ranged from 1 to 64 µM; dTPT3, ranged from 1 to 128 µM; dTAT1, ranged from 1 to 128 µM; dATP ranged from 15.6 to 1000 µM; dTTP, ranged from 16 to 1024 µM; dGTP and dCTP ranged from 2 to 1024 µM. The reaction lasted from 20 to 60 s for different nucleotides, then the reaction was quenched and analyzed as described above. The velocity at each nucleotide concentration was calculated by the incorporation efficiency per minute. GraphPad Prism 8 was used to plot velocities versus each nucleotide concentration, and the Michaelis–Menten equation was fitted to determine *V*_max_ and *K*_m_. All assays are independently repeated three times and values are the averages and standard deviations of three replicates.

### Primer Extension Assays

To test the primer extension ability after nucleotide insertion, the primer extension and full‐length synthesis assays were conducted according to the methods we previously reported.^[^ ^10a^ ^]^ In the primer extension experiment, we completed the single nucleotide incorporation reaction at the same conditions as the pre‐steady‐state assay, then the next nucleotide was added to the same reaction system with the final concentration of 6 µM and reacted for another 30 s. The further extended products, called *n* + 2 bands, were analyzed by the same method; the efficiency of primer further extension was calculated by the percentage of *n* + 2 bands in the total elongation products. The assay was performed in three independent replicates.

For the full‐length synthesis assays, dNaM and all four natural nucleotides were added to initiate primer extension for 1 min, the concentration of dNaM and each natural nucleotide was 6 µM, and the assay conditions were the same as the pre‐steady‐state assay. Except for not adding dNaM, samples with all other conditions consistent with the above reaction system served as a control. The full‐length bands were analyzed as above. The full‐length synthesis efficiency was calculated as the percentage of full‐length bands. Values are the average and standard deviations of three independent experiments.

### Measurement of Melting Temperature

Melting temperatures of the 13‐bp DNA duplexes (2.5 µM) were determined in 1 × UltraSYBR Mixture (Cowin Biotech Co., Ltd, Jiangsu, China) with fluorescence dye SYBR Green I specially binding to duplexes.^[^ ^18^ ^]^ This was a Taq DNA Polymerase mixture for quantitative real‐time PCR. Melting curves were recorded with a CFX96 real‐time PCR system and Manager 3.1 software, monitoring from 30 to 65 °C at a heating rate of 0.5 °C min^–1^. Melting temperatures were determined by calculating the derivative of the melting curves. Data are the average of three or more experiments within ± 0.5 °C.

### Labeling of εC Lesions in DNA Template by PCR Amplification

A 63‐mer single‐stranded DNA template with an εC lesion at the 34th site (KRAS–εC) was prepared. The labeling PCR amplification of KRAS–εC was conducted in a 25 µL reaction system with 12.5 µL OneTaq 2 × Master Mix, 0.2 ng KRAS–εC, 100 µM dTPT3TP, and dNaMTP, 0.4 µM forward (KRP‐F) and reverse primer (KRP‐R). The PCR underwent the following thermal cycling conditions: 96 °C for 3 min, (96 °C for 10 s, 50 °C for 15 s, 68 °C for 1 min) × 20, and a final extension at 68 °C for 5 min. The products were analyzed on a 2% agarose gel and extracted for further experiments. For Sanger sequencing, the primer was replaced with poly‐T primer (KRP‐dF, KRP‐dR).

### Bridge‐Base PCR

For further analysis, the labeling products were sent to Bridge‐base PCR for a second amplification.^[^ ^13^ ^]^ In Bridge‐base PCR, dTPT3TP was replaced with disoTATTP or absent, the number of cycles was adjusted to 36 cycles, and other conditions were the same as labeling PCR. It should be noted that for the sequencing of the εC lesions in the plasmid, the bridge‐base PCR was performed in the presence of dNaMTP only.

### Sequence Dependence Analysis

For sequence dependence analysis, a 79 mer single‐stranded DNA template with three random sites upstream and downstream around the εC lesion at the 36th site (cxwc‐εC) was prepared. We performed the labeling PCR according to the conditions described above with primer FendT‐F and FendT‐R. PCR products were extracted and then used as templates for the bridge‐base PCR with disoTATTP and dNaMTP. The final products were subjected to deep sequencing.

Deep sequencing was completed by Sangon Biotech (Shanghai, China). Briefly, Illumina library preparation was performed by PCR. The reaction system was set up as follows: 20 ng DNA template, 10 pmol P7 primer with index, 10 pmol P5 primer with index, and 2 × PCR Ready Mix 15 µL were mixed, and the final volume was adjusted to 30 µL using ribozyme‐free water, under the PCR conditions: 98 °C for 5 min, (94 °C for 30 s, 55 °C for 20 s, 72 °C for 30 s) × 5 cycles, final extension at 72 °C for 5 min. Then AMPure XP Beads were used to purify PCR products, and purified PCR products were subject to paired‐end sequencing on the NovaSeq6000/MiSeq. Raw reads were filtered by cutadapt (v 1.2.1), PRINSEQ‐lite (v 0.20.3), and usearch software (v 11.0.667), the remaining clean data were used for further analysis. A Python script was used to pick up each unique sequence and calculate the unique sequence and total sequences. The abundance and distribution of unique sequences were analyzed and illustrated by Microsoft Excel and GraphPad Prism 8.

### Enrichment of εC Lesions Based on Biotin‐Streptavidin System

To evaluate the enriched ability of dNaM with low abundance εC lesions, we conducted an enriched experiment based on biotin–streptavidin. The DNA template (KRAS–εC) was diluted by 100 times native KRAS to make the abundance of εC 1:100 (εC containing template: natural template), or diluted by 100 times native KRAS and excessive genomic DNA fragments to make the abundance of εC 1:1000. The diluted KRAS–εC was subjected to the labeling PCR amplification as follows: diluted DNA template 20 (1:100) or 200 (1:1000) ng, OneTaq DNA polymerase 0.5 U, Deepvent DNA polymerase 0.06 U, dTPT3^biotin^ 3 µM, dNaM 50 µM, MgSO_4_ 0.8 mM, 0.4 mM dNTPs, forward primer 2 µM, and reverse primer 2.4 µM were in 1 × OneTaq reaction buffer of total volume 25 µL. The mixture underwent the following thermal conditions: 15 cycles of denaturing at 96 °C for 30 s, annealing at 55 °C for 10 s, and elongation at 68 °C for 4 min. The PCR products were purified to remove excess dTPT3^biotin^, and then the purified products were co‐incubated with 10 µg streptavidin at 37 °C for 30 min. 6% nondenaturing PAGE was used to detect biotinylated dsDNA based on streptavidin‐induced electrophoretic mobility shift. TPT3^biotin^‐marked dsDNA was enriched and pulled down using Dynabeads MyOne Streptavidin C1 according to the manufacturer's instructions. Enriched biotinylated dsDNA was subject to a second PCR in the presence of dTPT3 and dNaM and then sent for Sanger sequencing.

### Alkylation of Plasmid DNA In Vitro and In Vivo Induced by Chloroacetaldehyde

To induce εC lesions in the plasmid DNA, Chloroacetaldehyde (CAA) was used to interfere with DNA in vitro or in vivo according to the literature.^[^ ^14^, ^15^ ^]^ For the in vitro experiment, 40 µg pUC‐19 plasmid extracted from *E. coli* DH5α cells was added to Chloroacetaldehyde aqueous solution (1.5 mM, 2 mL), and exposed at 37 °C for 24 h. After 24 h, the plasmid DNA was purified using Plasmid Mini‐Preps Kit. Partial plasmid DNA was digested to nucleosides using a Nucleoside Digestion Mix following the manufacturer's recommendation. Then the digestive products were subjected to HPLC analysis (UItimate 3000 system, Thermo SCIENTIFIC) equipped with a C18 analytical column (4.6 × 250 mm, Agilent Technologies). 0.1 M TEAB with a gradient of acetonitrile was used as the mobile phase, the gradient of acetonitrile was programmed as follows: 0–20%: 0–40 min; 20–80%: 40–50 min; decreased to 0% within 1 min and held for 8 min; flow rate, 1.0 mL min^−1^; detection wavelength, 272 nm. The suspicious peaks were collected for mass spectrometry. For the in vivo experiment, *E.coli* DH5α cell containing pUC‐19 plasmid was allowed to grow to 0.8 (OD_600_) in LB medium. Subsequently, CAA was added to the LB medium at the final concentration of 83 µM. The *E.coli* DH5α cell was allowed to grow for another 2 h, then the pUC‐19 plasmid was extracted using Plasmid Mini‐Preps Kit. Partial plasmids were digested using the same method and sent for HPLC and MS to monitor the occurrence of εC.

### Sequencing of DNA Damage In Vitro and In Vivo

The in vitro and in vivo damaged plasmids were also subjected to labeling PCR amplification using ten pairs of primers covering the whole plasmid under the same conditions. The labeled DNA was enriched and isolated as described above. Then the isolated DNA was sent to bridge‐base PCR with dNaMTP for a second amplification, and the products were purified and sent to Sangon Biotech (Shanghai, China) for deep sequencing assays. Briefly, the products were amplified using 2 × PCR Ready Mix for the preparation of the Illumina library and purified by Hieff NGS DNA Selection Beads, and the libraries were quantified and pooled. Then, the sequencing was performed with a Miseq Reagent Kit 3. Clean data was filtered as above and sequences were aligned with the target sequence (BWA 0.7.16). Finally, mutation frequencies at each site were calculated (GATK 4.x). The conversion ratios of unique sequences were analyzed and illustrated by Microsoft Excel and GraphPad Prism 8. The values calculated were defined by the ratio value of the mutation on the specific site of damaged samples to the mutation on the same site of referenced undamaged samples, and values > 3 were set down as lesion sites.

The damaged plasmids in vitro and in vivo were also subjected to Sanger sequencing and biotin–streptavidin‐mobility shift to verify the occurrence of εC in plasmid DNA. The plasmid DNA was subjected to labeling PCR amplification using another four pairs of primers under the same conditions described in “Enrichment of εC lesions based on biotin–streptavidin enrichment system,” εC lesions could be labeled by dNaM and then transformed to dTPT3^biotin^/dNaM pair during the continuous amplification process. One part of the products was used to enrich the labeled DNA using Dynabeads MyOne Streptavidin C1 according to the manufacturer's method. Enriched DNA was sent for a second amplification with dNaMTP and dTPT3TP, and products were sent for Sanger sequencing (Sangon Biotech, Shanghai, China). Then, other parts of the products were co‐incubated with 10 µg streptavidin at 37 °C for 30 min, and detected by 6% nondenaturing PAGE electrophoresis based biotin–streptavidin‐mobility shift.

### Statistical Analyses

Fluorescence intensities of incorporation and extension assays were quantified by Amersham Imager 600 Analysis Software. The abundance and distribution of unique sequences in sequence dependence analysis were analyzed and illustrated by Microsoft Excel and GraphPad Prism 8. Mutation frequencies at each site were calculated (GATK 4.x). The conversion ratios of unique sequences were analyzed and illustrated by Microsoft Excel and GraphPad Prism 8. The values calculated were defined by the ratio value of the mutation on the specific site of damaged samples to the mutation on the same site of referenced undamaged samples, and values > 3 were set down as lesion sites. All assays are independently repeated three times and values are the averages or averages and standard deviations of three replicates.

## Conflict of Interest

The authors declare no conflict of interest.

## Author Contributions

H.W., W.T., and W.Z. contributed equally to this work. L.L., Z.A., and H.W. conceived the project. H.W., W.T., W.A., S.W., R.Z., B.Y., and J.Z. performed the experiments. H.W., W.T., and J.D. analyzed the data. L.L. and H.W. wrote the initial manuscript. All authors reviewed and edited the manuscript. L.L. supervised the work and acquired funding.

## Supporting information

Supporting Information

Supplemental Tables

## Data Availability

The data that support the findings of this study are openly available in National Center for Biotechnology Information at https://www.ncbi.nlm.nih.gov/bioproject, reference number 16.

## References

[1] a) B. Singer , Nature 1976, 264, 333; 1004554 10.1038/264333a0

[2] a) A. K. Basu , J. M. Essigmann , Chem. Res. Toxicol. 2022, 35, 1655; 35881568 10.1021/acs.chemrestox.2c00155 PMC10201539

[3] a) M. H. Liu , B. M. Costa , E. C. Bianchini , U. Choi , R. C. Bandler , E. Lassen , M. Gronska‐Peski , A. Schwing , Z. R. Murphy , D. Rosenkjaer , S. Picciotto , V. Bianchi , L. Stengs , M. Edwards , N. M. Nunes , C. A. Loh , T. K. Truong , R. E. Brand , T. Pastinen , J. R. Wagner , A. B. Skytte , U. Tabori , J. E. Shoag , G. D. Evrony , Nature 2024; 10.1038/s41586-024-07532-8 PMC11216816 38867045

[4] A. Barbin , H. Bartsch , P. Leconte , M. Radman , Nucleic Acids Res. 1981, 9, 375. 7010314 10.1093/nar/9.2.375 PMC326699

[5] a) D. W. Roberts , M. I. Churchwell , F. A. Beland , J. L. Fang , D. R. Doerge , Anal. Chem. 2001, 73, 303; 11199982 10.1021/ac000866n

[6] J. C. Delaney , L. Smeester , C. Y. Wong , L. E. Frick , K. Taghizadeh , J. S. Wishnok , C. L. Drennan , L. D. Samson , J. M. Essigmann , Nat. Struct. Mol. Biol. 2005, 12, 855. 16200073 10.1038/nsmb996

[7] M. Moriya , W. Zhang , F. Johnson , A. P. Grollman , Proc. Natl. Acad. Sci. USA 1994, 91, 11899. 7991554 10.1073/pnas.91.25.11899 PMC45343

[8] I. A. Chaim , A. Gardner , J. E. Wu , T. Iyama , D. M. Wilson , L. D. Samson , Nucleic Acids Res. 2017, 45, 3242. 28115629 10.1093/nar/gkx015 PMC5389632

[9] a) S. Hoshika , N. A. Leal , M. J. Kim , M. S. Kim , N. B. Karalkar , H. J. Kim , A. M. Bates , N. E. Watkins , H. A. SantaLucia , A. J. Meyer , S. DasGupta , J. A. Piccirilli , A. D. Ellington , J. SantaLucia , M. M. Georgiadis , S. A. Benner , Science 2019, 363, 884; 30792304 10.1126/science.aat0971 PMC6413494

[10] a) W. Zhu , H. Wang , X. Li , W. Tie , B. Huo , A. Zhu , L. Li , J. Am. Chem. Soc. 2022, 144, 20165; 36287063 10.1021/jacs.2c06110

[11] a) Z. Yang , A. M. Sismour , P. Sheng , N. L. Puskar , S. A. Benner , Nucleic Acids Res. 2007, 35, 4238; 17576683 10.1093/nar/gkm395 PMC1934989

[12] H. Wang , L. Wang , N. Ma , W. Zhu , B. Huo , A. Zhu , L. Li , ACS Synth. Biol. 2022, 11, 334. 34889587 10.1021/acssynbio.1c00451

[13] H. Wang , W. Zhu , C. Wang , X. Li , L. Wang , B. Huo , H. Mei , A. Zhu , G. Zhang , L. Li , Nucleic Acids Res. 2023, 51, e52. 36971131 10.1093/nar/gkad218 PMC10201413

[14] P. Kowalczyk , J. M. Cieśla , M. Saparbaev , J. Laval , B. Tudek , Acta Biochim. Pol. 2006, 53, 337. 16582987

[15] J. Jia , S. Q. Chen , W. Z. Pan , S. N. Yu , X. T. Zhao , Y. Hao , Y. M. Shen , Y. Cheng , C. L. Wei , F. J. Tian , X. Y. Yan , Y. L. Qiu , J. Appl. Toxicol. 2022, 42, 490. 34601724 10.1002/jat.4234

[16] S. S. Pujari , P. Leonard , F. Seela , J. Org. Chem. 2014, 79, 4423. 24693949 10.1021/jo500392j

[17] M. F. Goodman , S. Creighton , L. B. Bloom , J. Petruska , Crit. Rev. Biochem. Mol. Biol. 1993, 28, 83. 8485987 10.3109/10409239309086792

[18] R. Cruz‐Flores , H. N. Mai , A. K. Dhar , Mol. Cell. Probes 2019, 43, 20. 30576786 10.1016/j.mcp.2018.12.004 PMC7127373

