# Supplementary material for: Recognition and Sequencing of Mutagenic DNA Adduct at Single‐Base Resolution Through Unnatural Base Pair
Source: Adv Sci (Weinh). 2024 Sep 3;11(40):2404622. doi: 10.1002/advs.202404622 (PMC11515917; doi:10.1002/advs.202404622)
Supplement: Supplementary file 1 — Supporting Information [file ADVS-11-2404622-s001.docx]

Supporting Information：

**Recognition and Sequencing of Mutagenic DNA Adduct at Single-Base Resolution through Unnatural Base Pair**

Honglei Wang, Wenchao Tie, Wuyuan Zhu, Shuyuan Wang, Ruzhen Zhang, Jianlin Duan, Bingyu Ye, Anlian Zhu, and Lingjun Li*

Henan Key Laboratory of Organic Functional Molecule and Drug Innovation, Collaborative Innovation Center of Henan Province for Green Manufacturing of Fine Chemicals, School of Chemistry and Chemical Engineering, Key Laboratory of Green Chemical Media and Reactions, Ministry of Education, Henan Normal University, Xinxiang, Henan, 453007, China. ^2^State Key Laboratory of Antiviral Drug and Pingyuan Lab, Henan Normal University, Xinxiang, Henan, 453007, China.

**Table of Contents**

[1. Synthesis procedure of εC phosphoramidite building block 1](#_Toc173743637)

[2. NMR Spectra 3](#_Toc173743638)

[3. Sequence used in this study 6](#_Toc173743639)

[4. Supplementary Figures 9](#_Toc173743640)

[5. Reference 19](#_Toc173743641)

# Synthesis procedure of εC phosphoramidite ****building block****

**Scheme S1**. (a) 37^o^C 24 h, PH = 4.5-5.0; (b) DMT-Cl, anhydrous pyridine, room temperature, 3 h; (c) DIPEA, CEP-Cl, dry CH_2_Cl_2_, room temperature, a nitrogen atmosphere, 1 h.

Compound **a.** Deoxycytidine (0.5 g, 2.2 mmol) was dissolved in 40% chloroacetaldehyde aqueous solution (6.5 mL). Sodium acetate was added to maintain the pH in the range of 4.5-5. The mixture was reacted at 37^o^C for 24 h. After vacuum drying, compound **a** (300mg, 54%) was obtained with 5% MeOH/CH_2_Cl_2_ by column chromatography.

^1^H NMR (400 MHz, D_2_O) δ 7.50 (d, *J* = 1.2 Hz, 1H), 7.41 (d, *J* = 8.0 Hz, 1H), 7.18 (d, *J* = 1.6 Hz, 1H), 6.52 (d, *J* = 8.0 Hz, 1H), 6.33 (t, *J* = 6.6 Hz, 1H), 4.43 – 4.36 (m, 1H), 3.96 (dd, *J* = 8.7, 3.8 Hz, 1H), 3.76 (dd, *J* = 12.4, 3.6 Hz, 1H), 3.68 (dd, *J* = 12.4, 5.1 Hz, 1H), 2.41 – 2.32 (m, 1H), 2.31 – 2.22 (m, 1H). ^13^C NMR (100 MHz, MeOD) δ 145.74, 145.43, 131.29, 128.75, 112.59, 97.56, 87.84, 86.09, 70.87, 61.48, 40.26. HRMS (ESI^+^) m/z calcd for C_11_H_14_N_3_O_4_^+^ (M+H^+^), 252.0979, found, 251.0977.

Compound **b.** **a** (300 mg, 1.2 mmol) and 4,4'-Dimethoxytrityl chloride (DMT-Cl, 1.44 mmol) were dissolved in anhydrous pyridine (6 mL), and then the mixture was reacted at room temperature for 3 h under a nitrogen atmosphere, monitored by TLC, quenched with methanol, and finally evaporated under reduced pressure. The product (360 mg, 55%) was obtained with 2% MeOH/CH_2_Cl_2_ by column chromatography.

^1^H NMR (400 MHz, MeOD) δ 7.75 (d, *J* = 8.0 Hz, 1H), 7.74 (dd, *J* = 8.3, 4.7 Hz, 2H), 7.73 (d, *J* = 1.3 Hz, 1H), 7.46 – 7.38 (m, 2H), 7.44 – 7.39 (m, 2H), 7.35 – 7.12 (m, 8H), 7.34 – 7.15 (m, 8H), 6.82 (d, *J* = 8.9 Hz, 4H), 6.82 (d, *J* = 8.9 Hz, 4H), 6.47 (t, *J* = 6.3 Hz, 1H), 6.47 (t, *J* = 6.3 Hz, 1H), 6.24 (d, *J* = 8.0 Hz, 1H), 6.24 (d, *J* = 8.0 Hz, 1H), 4.72 – 4.53 (m, 1H), 4.61 – 4.55 (m, 1H), 4.05 (q, *J* = 3.4 Hz, 1H), 4.05 (q, *J* = 3.4 Hz, 1H), 3.72 (s, 6H), 3.72 (s, 6H), 3.44 (d, *J* = 3.4 Hz, 2H), 3.44 (d, *J* = 3.4 Hz, 2H), 2.40 (ddd, *J* = 20.0, 10.0, 6.0 Hz, 2H), 2.40 (ddd, *J* = 20.0, 10.0, 6.0 Hz, 2H). ^13^C NMR (100 MHz, MeOD) δ 158.85, 145.56, 145.34, 144.66, 135.54, 135.34, 131.31, 130.04, 128.74, 128.06, 127.51, 126.64, 112.81, 112.60, 97.39, 86.76, 86.55, 86.14, 70.48, 62.92, 54.35, 40.62. HRMS (ESI^+^) m/z calcd for C_32_H_32_N_3_O_6_^+^ (M+H^+^) 554.2286, found 554.2285.

Compound **C. b** (360 mg) was dried with anhydrous toluene by coevaporations and then resolved in dry CH_2_Cl_2_ (2 mL). N,N-diisopropylethylamine (DIPEA, 0.28 mL, 1.63 mmol) and 2-cyanoethyl-N,N-diisopropyl chlorophosphoramidite (CEP-Cl, 0.19 mL, 0.85 mmol) were added in turn. The reaction was stirred at room temperature for 90 min under a nitrogen atmosphere. Then Compound C (350 mg, 46%) was obtained with 50% ethyl acetate/hexane after drying. ^31^P NMR (162 MHz, MeOD) δ 148.51 (s).

# NMR Spectra

**6-((2R,4S,5R)-4-hydroxy-5-(hydroxymethyl)tetrahydrofuran-2-yl)imidazo[1,2-c]pyrimidin-5(6H)-one**

400 MHz ^1^H NMR spectrum of **Compound a** (D_2_O)

100 MHz ^13^C NMR spectrum of **Compound a** (Methanol-*d_4_*)


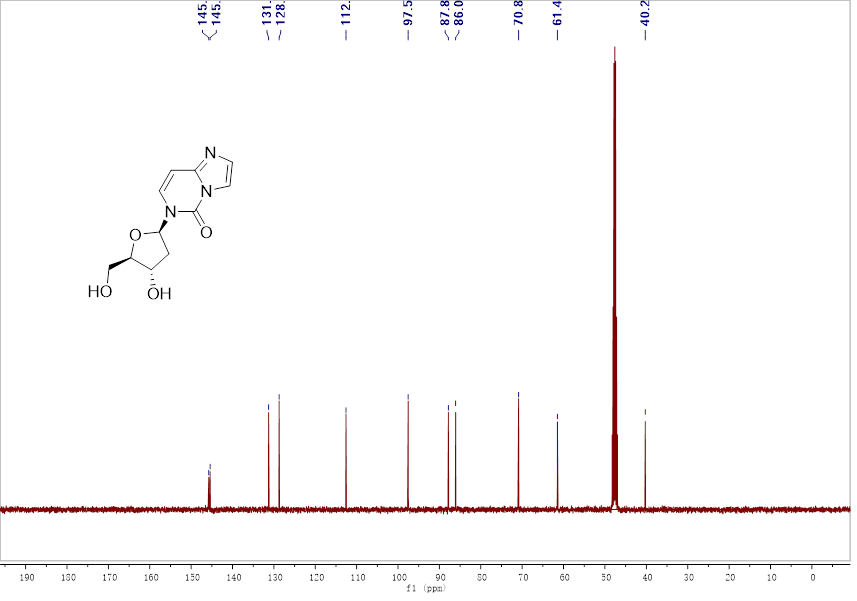


**6-(5-((bis(4-methoxyphenyl)(phenyl)methoxy)methyl)-4-hydroxytetrahydrofuran-2-yl)imidazo[1,2-c]pyrimidin-5(6H)-one**

400 MHz ^1^H NMR spectrum of **Compound b** (Methanol-*d_4_*)


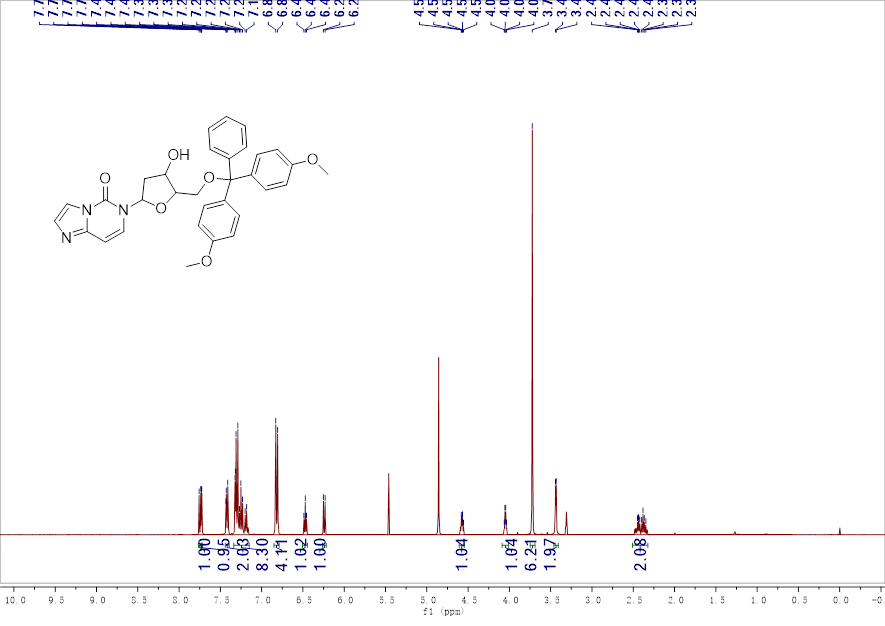


100 MHz ^13^C NMR spectrum of **Compound b** (Methanol-*d_4_*)


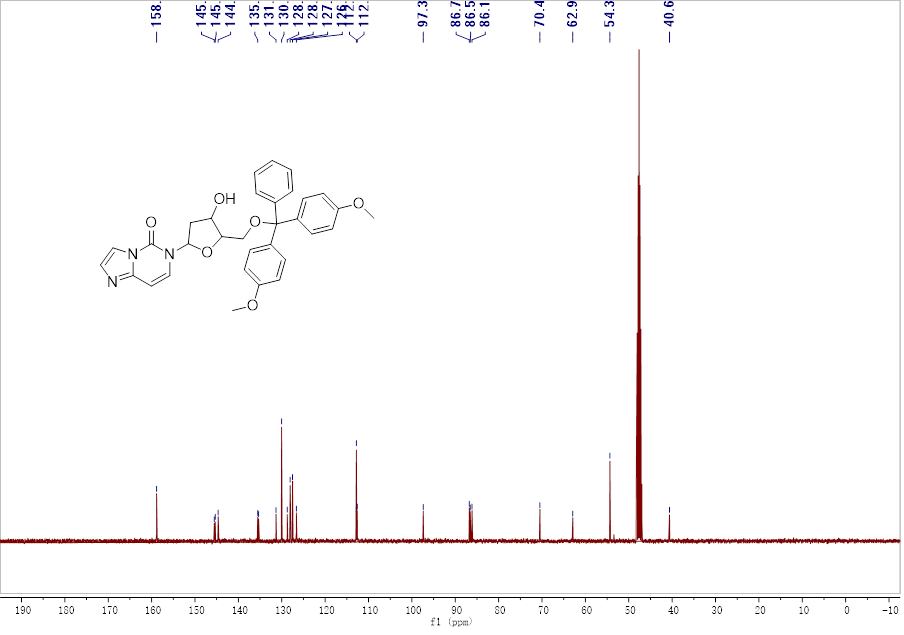


**2-((bis(4-methoxyphenyl)(phenyl)methoxy)methyl)-5-(5-oxoimidazo[1,2-c]pyrimidin-6(5H)-yl)tetrahydrofuran-3-yl (2-cyanoethyl) diisopropylphosphoramidite**

162 MHz ^31^P NMR spectrum of **Compound c** (Methanol-*d_4_*)


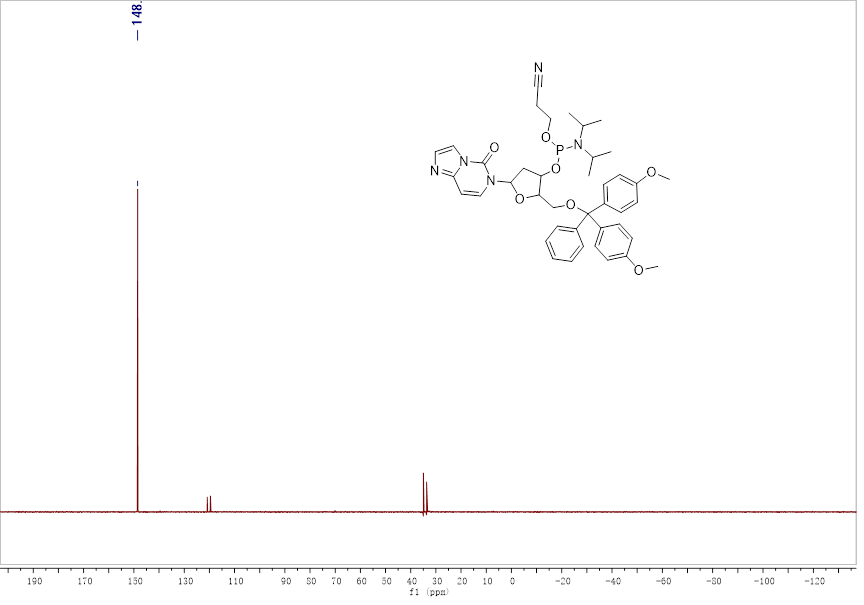


# Sequence used in this study

Table S1. Sequence used in this study(5’→3’)

| Primer and template for kinetic assay | |
| --- | --- |
| DL-Primer | HEX-TAATACGACTCACTATAGGGAGA |
| DL-εC-temp | TCAC/εC/TCTCCCTATAGTGAGTCGTATTA |
| Primer and template for sequence dependence assay | |
| cxwc-εC | CACACAGGAAACAGCTATGACTGAATTCACCANNN/εC/NNNAACTCTTGTTAAGCTTCCTATAGTGAGTCGTATTAATTTC |
| FendT-F | TTTTTTTTTTTTTTTTTTTTTTTTTTTTTTTTTTTTTTTTTTTTTTTTTTTTTTTTTTTCACACAGGAAACAGCTAT |
| FendT-R | TTTTTTTTTTTTTTTTTTTTTTTTTTTTTTTTTTTTTTTTTTTTTTTTTTTTTTTTTTTTTGAAATTAATACGACTCACTATAGG |
| Primer and template for PCR analysis | |
| KRAS-εC | CAGGAAACAGCTATGACACTCTTGCCTACGCCA/εC/CAGCTCCAACTACACTGGCCGTCGTTTTAC |
| KRP-F | CAGGAAACAGCTATGAC |
| KRP-R | GTAAAACGACGGCCAGT |
| KRP-dF | TTTTTTTTTTTTTTTTTTTTTTTTTTTTTTTTTTTTTTTTTTTTTTTTTTTTTTTTTTTTTCAGGAAACAGCTATGAC |
| KRP-dR | TTTTTTTTTTTTTTTTTTTTTTTTTTTTTTTTTTTTTTTTTTTTTTTTTTTTTTTTTTTTTGTAAAACGACGGCCAGT |
| Primer for testing the εC site in the plasmids by Sanger sequencing | |
| PUC-1F | TCACTGGCCGTCGTTTTACA |
| PUC-1R | CCGGCGTCAATACGGGATAA |
| PUC-2F | TTCCGTGTCGCCCTTATTCC |
| PUC-2R | CTGACGCTCAGTGGAACGAA |
| PUC-3F | ATCTACACGACGGGGAGTCA |
| PUC-3R | CCGCTTACCGGATACCTGTC |
| PUC-4F | AGTTACCGGATAAGGCGCAG |
| PUC-4R | CGTTGTAAAACGACGGCCAG |
| Primer for detecting the εC site in the plasmids by deep sequencing | |
| 1F | TCACTGGCCGTCGTTTTACA |
| 1R | ACCTCTGACACATGCAGCTC |
| 2F | CAGACAAGCTGTGACCGTCT |
| 2R | CTCGTGCACCCAACTGATCT |
| 3F | ACCCAGAAACGCTGGTGAAA |
| 3R | TCCGGTTCCCAACGATCAAG |
| 4F | ACACTGCGGCCAACTTACTT |
| 4R | CAGTGCTGCAATGATACCGC |
| 5F | TTCCGGCTGGCTGGTTTATT |
| 5R | TGACGCTCAGTGGAACGAAA |
| 6F | ATCGCTGAGATAGGTGCCTC |
| 6R | GGCGGTGCTACAGAGTTCTT |
| 7F | GCCGGATCAAGAGCTACCAA |
| 7R | CCGCTTACCGGATACCTGTC |
| 8F | TTGGAGCGAACGACCTACAC |
| 8R | CAGCTCACTCAAAGGCGGTA |
| 9F | GCTCACATGTTCTTTCCTGCG |
| 9R | CGTTGTAAAACGACGGCCAG |
| 10F | CACTCATTAGGCACCCCAGG |
| 10R | CTATGCGGCATCAGAGCAGA |
| Oligonucleotides for thermal stability | |
| C-ODN1A | CTTTCT/**A/**CTCCTT |
| C-ODN1T | CTTTCT/**T/**CTCCTT |
| C-ODN1G | CTTTCT/**G/**CTCCTT |
| C-ODN1C | CTTTCT/**C/**CTCCTT |
| C-ODN1εC | CTTTCT/**εC/**CTCCTT |
| C-ODN1N | CTTTCT/**NaM/**CTCCTT |
| C-ODN2A | AAGGAG/**A/**AGAAAG |
| C-ODN2T | AAGGAG/**T/**AGAAAG |
| C-ODN2G | AAGGAG/**G/**AGAAAG |
| C-ODN2C | AAGGAG/**C/**AGAAAG |
| C-ODN2εC | AAGGAG/**εC/**AGAAAG |
| C-ODN2N | AAGGAG/**NaM/**AGAAAG |
| C-ODN1UT | CTTTCT/**TPT3/**CTCCTT |
| C-ODN2UT | AAGGAG/**TPT3/**AGAAAG |
| Sequence of pUC-19 plasmid | |
| ATACGCAAACCGCCTCTCCCCGCGCGTTGGCCGATTCATTAATGCAGCTGGCACGACAGGTTTCCCGACTGGAAAGCGGGCAGTGAGCGCAACGCAATTAATGTGAGTTAGCTCACTCATTAGGCACCCCAGGCTTTACACTTTATGCTTCCGGCTCGTATGTTGTGTGGAATTGTGAGCGGATAACAATTTCACACAGGAAACAGCTATGACCATGATTACGCCAAGCTTGCATGCCTGCAGGTCGACTCTAGAGGATCCCCGGGTACCGAGCTCGAATTCACTGGCCGTCGTTTTACAACGTCGTGACTGGGAAAACCCTGGCGTTACCCAACTTAATCGCCTTGCAGCACATCCCCCTTTCGCCAGCTGGCGTAATAGCGAAGAGGCCCGCACCGATCGCCCTTCCCAACAGTTGCGCAGCCTGAATGGCGAATGGCGCCTGATGCGGTATTTTCTCCTTACGCATCTGTGCGGTATTTCACACCGCATATGGTGCACTCTCAGTACAATCTGCTCTGATGCCGCATAGTTAAGCCAGCCCCGACACCCGCCAACACCCGCTGACGCGCCCTGACGGGCTTGTCTGCTCCCGGCATCCGCTTACAGACAAGCTGTGACCGTCTCCGGGAGCTGCATGTGTCAGAGGTTTTCACCGTCATCACCGAAACGCGCGAGACGAAAGGGCCTCGTGATACGCCTATTTTTATAGGTTAATGTCATGATAATAATGGTTTCTTAGACGTCAGGTGGCACTTTTCGGGGAAATGTGCGCGGAACCCCTATTTGTTTATTTTTCTAAATACATTCAAATATGTATCCGCTCATGAGACAATAACCCTGATAAATGCTTCAATAATATTGAAAAAGGAAGAGTATGAGTATTCAACATTTCCGTGTCGCCCTTATTCCCTTTTTTGCGGCATTTTGCCTTCCTGTTTTTGCTCACCCAGAAACGCTGGTGAAAGTAAAAGATGCTGAAGATCAGTTGGGTGCACGAGTGGGTTACATCGAACTGGATCTCAACAGCGGTAAGATCCTTGAGAGTTTTCGCCCCGAAGAACGTTTTCCAATGATGAGCACTTTTAAAGTTCTGCTATGTGGCGCGGTATTATCCCGTATTGACGCCGGGCAAGAGCAACTCGGTCGCCGCATACACTATTCTCAGAATGACTTGGTTGAGTACTCACCAGTCACAGAAAAGCATCTTACGGATGGCATGACAGTAAGAGAATTATGCAGTGCTGCCATAACCATGAGTGATAACACTGCGGCCAACTTACTTCTGACAACGATCGGAGGACCGAAGGAGCTAACCGCTTTTTTGCACAACATGGGGGATCATGTAACTCGCCTTGATCGTTGGGAACCGGAGCTGAATGAAGCCATACCAAACGACGAGCGTGACACCACGATGCCTGTAGCAATGGCAACAACGTTGCGCAAACTATTAACTGGCGAACTACTTACTCTAGCTTCCCGGCAACAATTAATAGACTGGATGGAGGCGGATAAAGTTGCAGGACCACTTCTGCGCTCGGCCCTTCCGGCTGGCTGGTTTATTGCTGATAAATCTGGAGCCGGTGAGCGTGGGTCTCGCGGTATCATTGCAGCACTGGGGCCAGATGGTAAGCCCTCCCGTATCGTAGTTATCTACACGACGGGGAGTCAGGCAACTATGGATGAACGAAATAGACAGATCGCTGAGATAGGTGCCTCACTGATTAAGCATTGGTAACTGTCAGACCAAGTTTACTCATATATACTTTAGATTGATTTAAAACTTCATTTTTAATTTAAAAGGATCTAGGTGAAGATCCTTTTTGATAATCTCATGACCAAAATCCCTTAACGTGAGTTTTCGTTCCACTGAGCGTCAGACCCCGTAGAAAAGATCAAAGGATCTTCTTGAGATCCTTTTTTTCTGCGCGTAATCTGCTGCTTGCAAACAAAAAAACCACCGCTACCAGCGGTGGTTTGTTTGCCGGATCAAGAGCTACCAACTCTTTTTCCGAAGGTAACTGGCTTCAGCAGAGCGCAGATACCAAATACTGTTCTTCTAGTGTAGCCGTAGTTAGGCCACCACTTCAAGAACTCTGTAGCACCGCCTACATACCTCGCTCTGCTAATCCTGTTACCAGTGGCTGCTGCCAGTGGCGATAAGTCGTGTCTTACCGGGTTGGACTCAAGACGATAGTTACCGGATAAGGCGCAGCGGTCGGGCTGAACGGGGGGTTCGTGCACACAGCCCAGCTTGGAGCGAACGACCTACACCGAACTGAGATACCTACAGCGTGAGCTATGAGAAAGCGCCACGCTTCCCGAAGGGAGAAAGGCGGACAGGTATCCGGTAAGCGGCAGGGTCGGAACAGGAGAGCGCACGAGGGAGCTTCCAGGGGGAAACGCCTGGTATCTTTATAGTCCTGTCGGGTTTCGCCACCTCTGACTTGAGCGTCGATTTTTGTGATGCTCGTCAGGGGGGCGGAGCCTATGGAAAAACGCCAGCAACGCGGCCTTTTTACGGTTCCTGGCCTTTTGCTGGCCTTTTGCTCACATGTTCTTTCCTGCGTTATCCCCTGATTCTGTGGATAACCGTATTACCGCCTTTGAGTGAGCTGATACCGCTCGCCGCAGCCGAACGACCGAGCGCAGCGAGTCAGTGAGCGAGGAAGCGGAAGAGCGCCCA | |

# Supplementary Figures


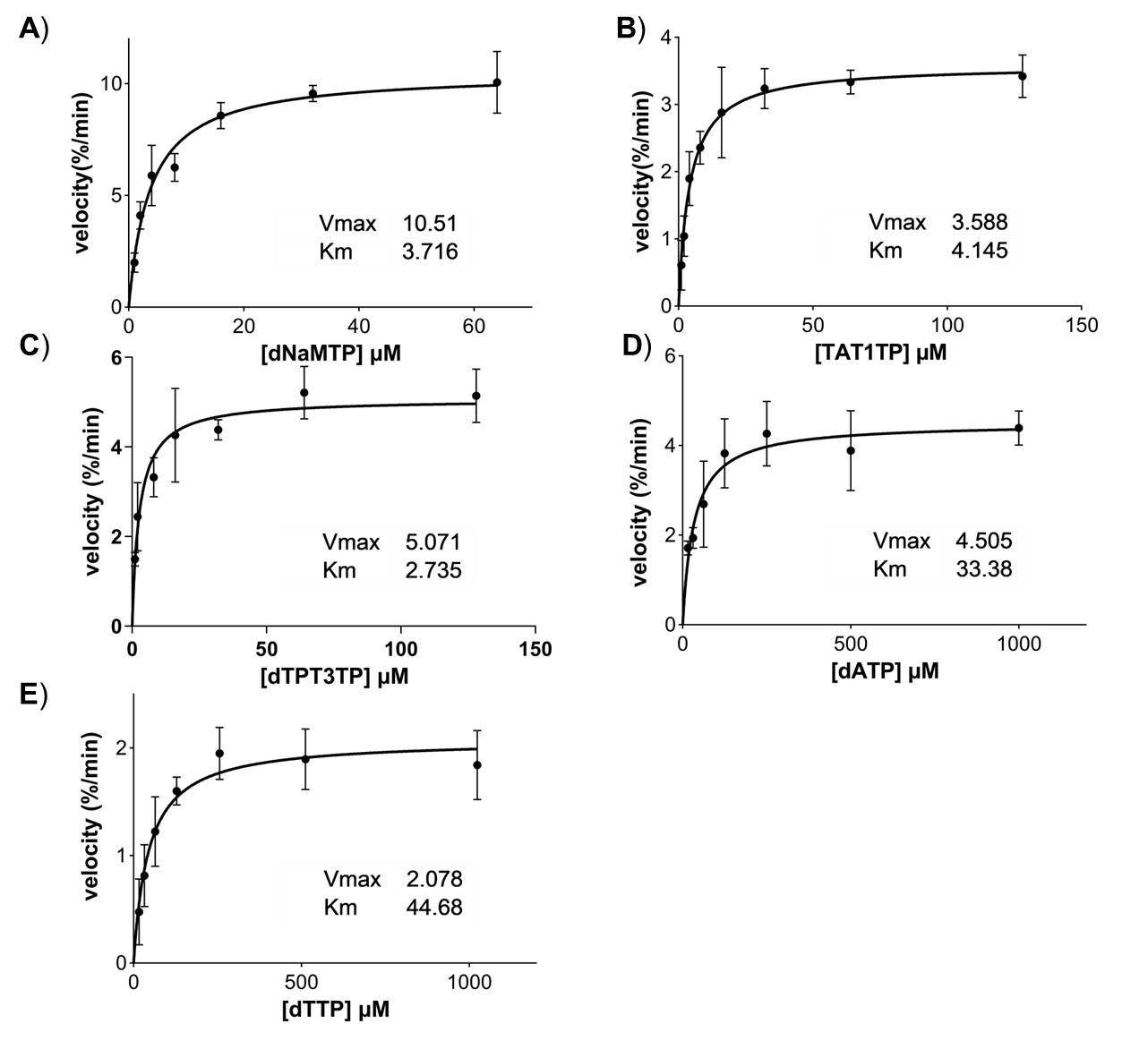


Figure S1. Michaelis-Menten curves. The Michaelis-Menten curve for inserting dNaMTP (A), TAT1TP (B), dTPT3TP (C), dATP (D), and dTTP (E) opposite the εC site in templates. Values are means ± SD (n=3).


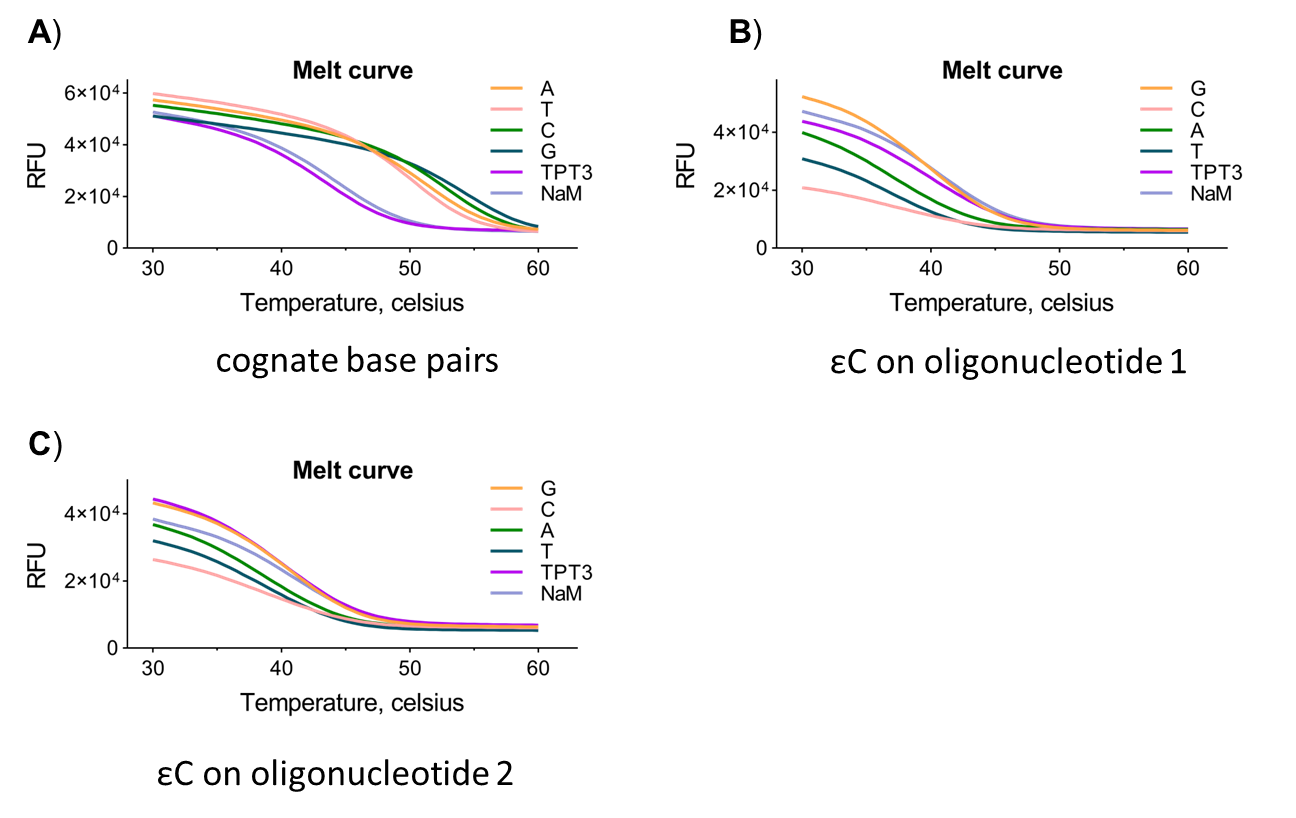


Figure S2. Melting curves. Melting curves of duplex fragments with cognate base pairs (A) or εC on either strand (B, εC on oligonucleotide 1; C, εC on oligonucleotide 2).


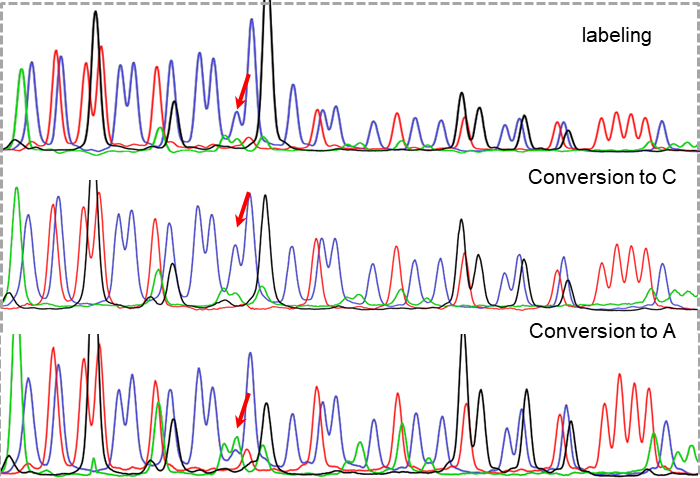


Figure S3. Sanger sequencing of the labeling and conversion products. The sense stands with TPT3 or original εC are shown. Due to the sequence content, the sense stands with TPT3 is particularly read-through, the red arrowheads indicate the corresponding εC location.


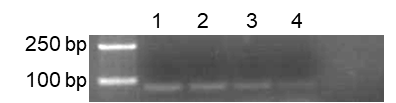


Figure S4. Labeling PCR with 20, 2, 1, and 0.1 pg εC template, samples 1 to 4.

Figure S5. The distribution frequency of dinucleotides surrounding the εC site after conversion, red squares represent dinucleotides downstream, blue circulars represent dinucleotides upstream, the coordinate unit is 10 000.


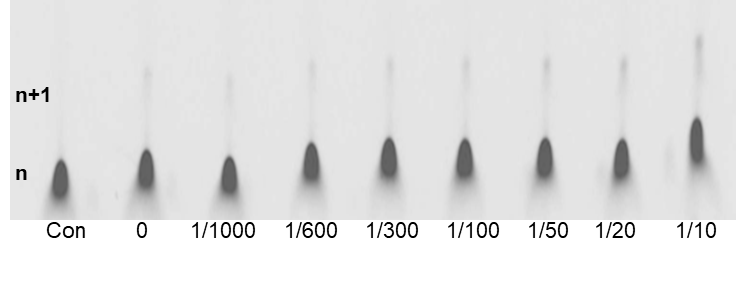


Figure S6. Representative gel of dNaMTP incorporation with various dilutions of the εC template and a constant concentration of dC template.


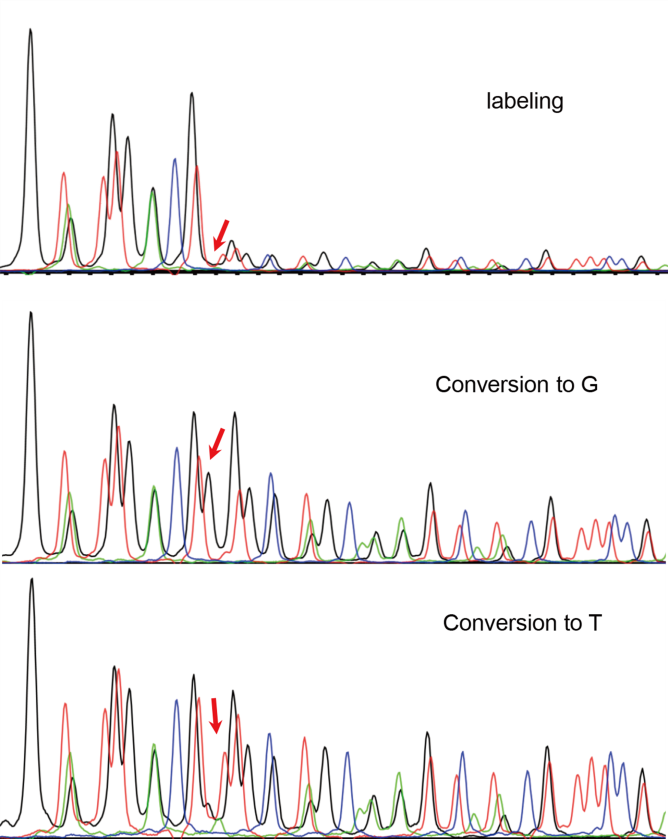


**Figure** S7. Sanger sequencing of the labeling and conversion products after enrichment with an abundance level of 1: 1000, only the antisense strands with NaM are shown, the red arrowheads indicate the corresponding εC location with signal termination or conversion to G or T.


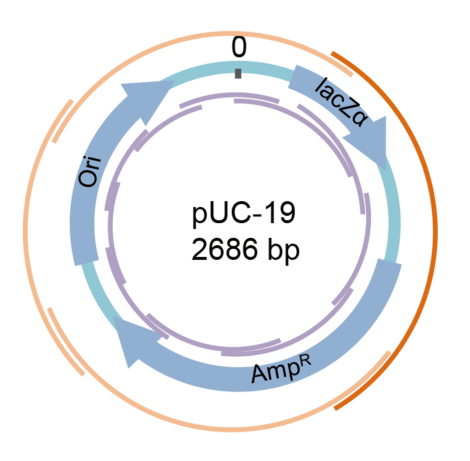


**Figure** S8. pUC-19 plasmid profile with primers outside (orange) for Sanger terminal sequencing and inside for deep sequencing, deep orange indicates the representative fragment of primer PUC-1.


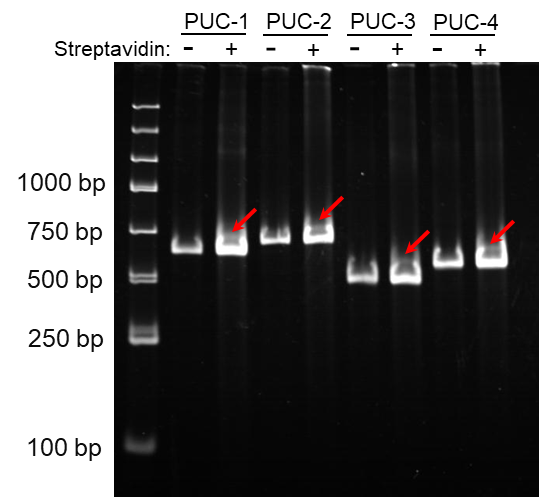


Figure S9. Representative gel for biotin-streptavidin-based strand shift of the εC lesion in the pUC-19 plasmid exposed to CAA. The shift bands are marked with red arrowheads.


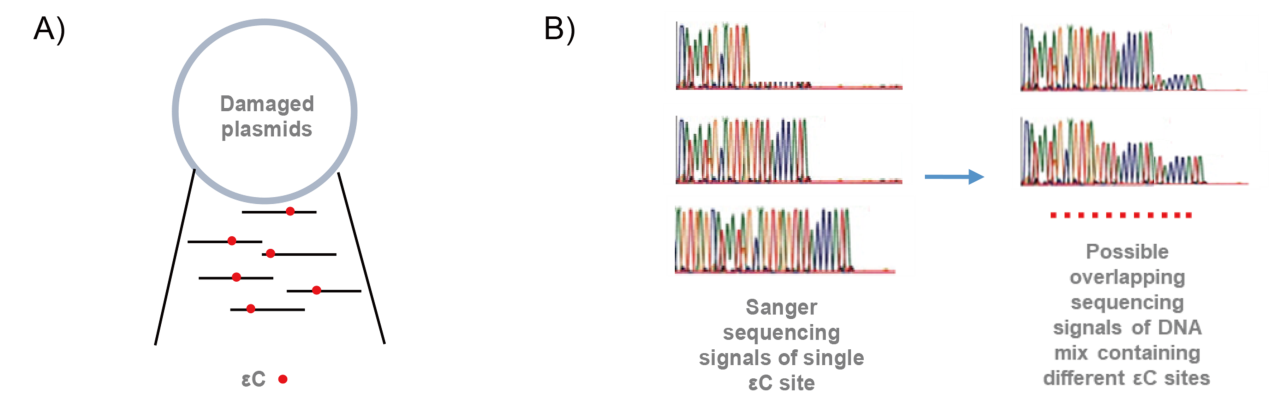


**Figure** S10. The schematic diagram of εC distribution and overlapping sequencing signals. (A) possible characteristic of εC distribution in the damaged DNA fragments of pUC-19 plasmid, εC may distributed at different loci. (B) The scheme of overlapping sequencing signals of DNA mix containing different εC sites.


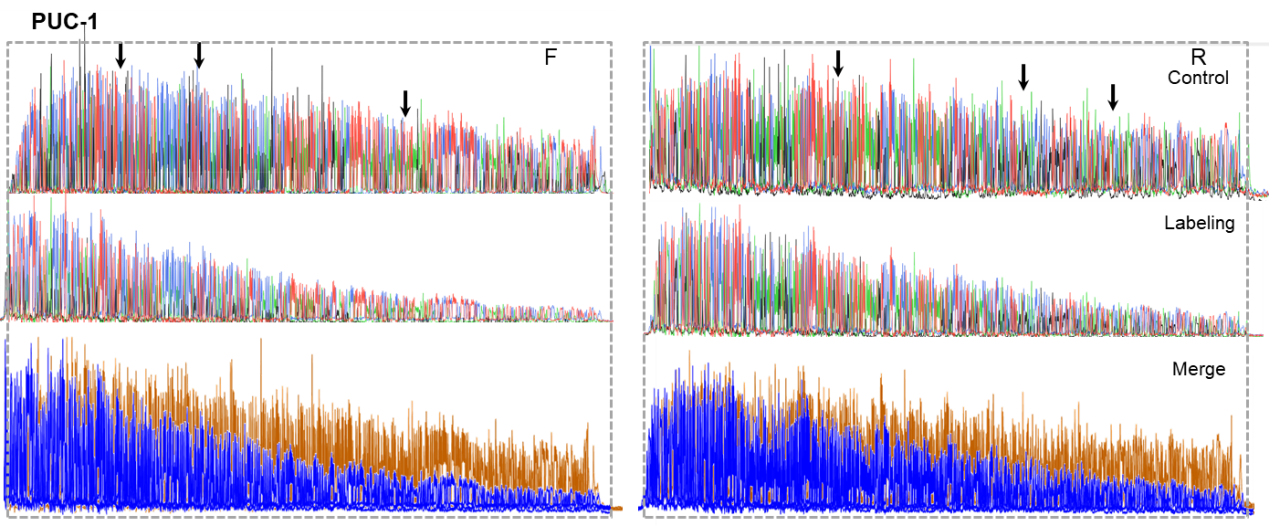


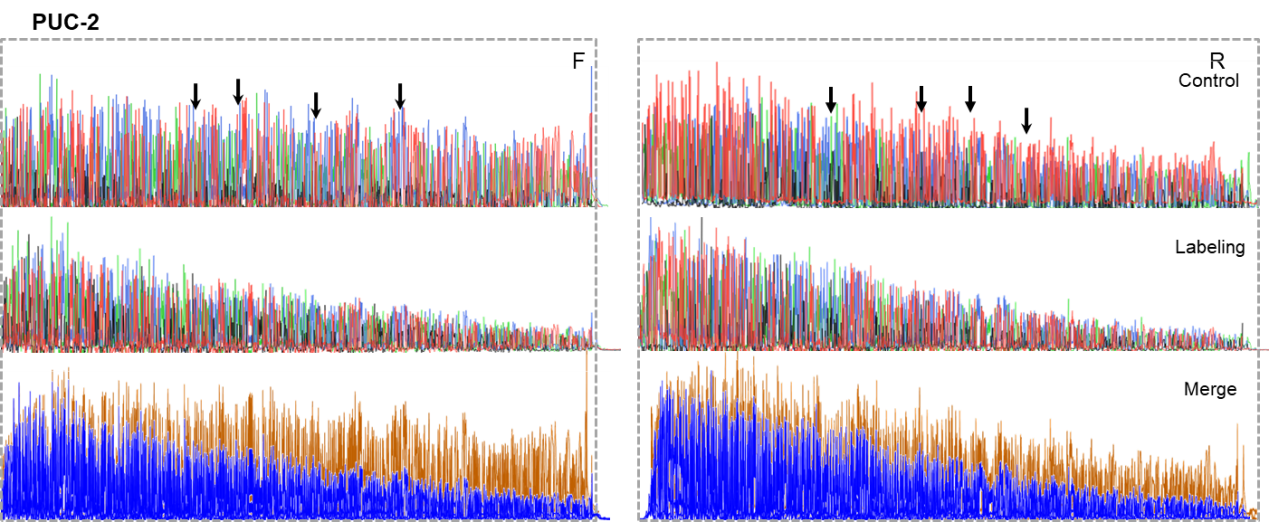


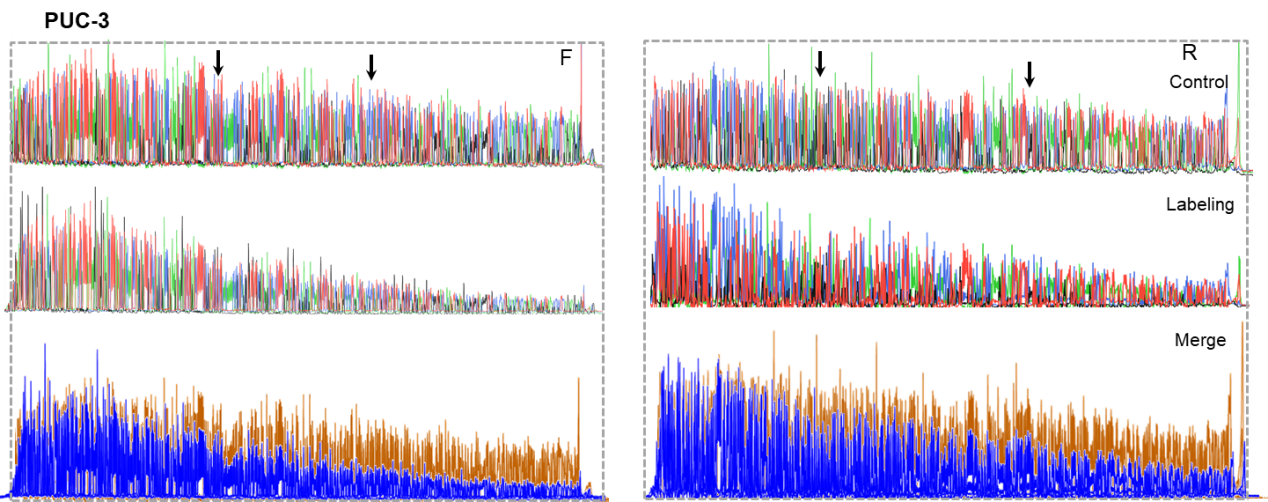


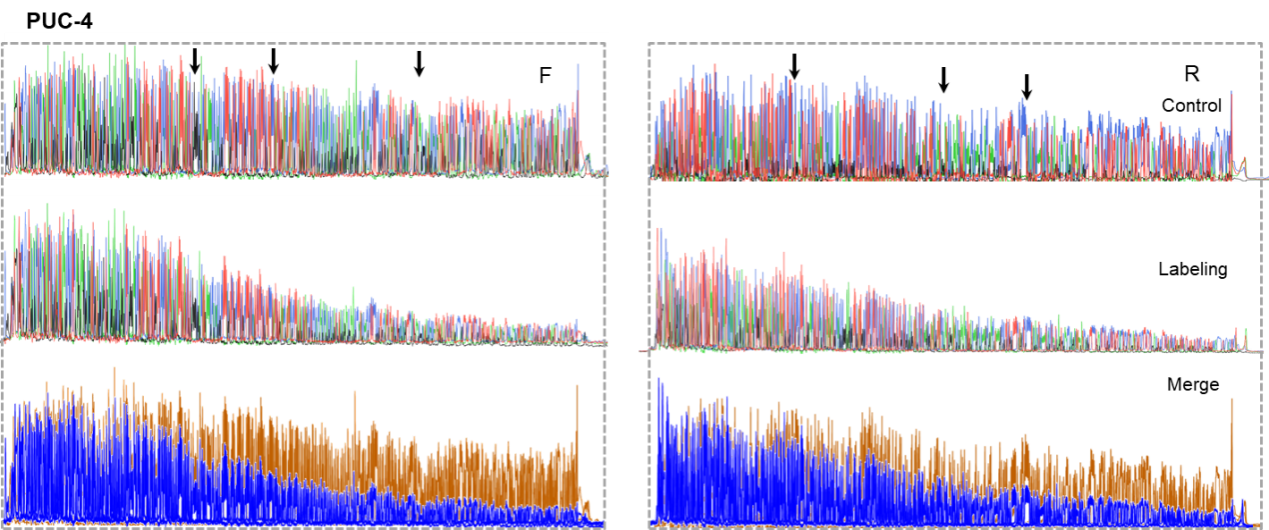


Figure S11. Sequencing of εC lesions in the pUC-19 plasmids exposed to CAA using primers PUC-1-4. Sense (F)and anti-sense (R) strands were both shown. Black arrowheads indicate regions with strong signal attenuation.


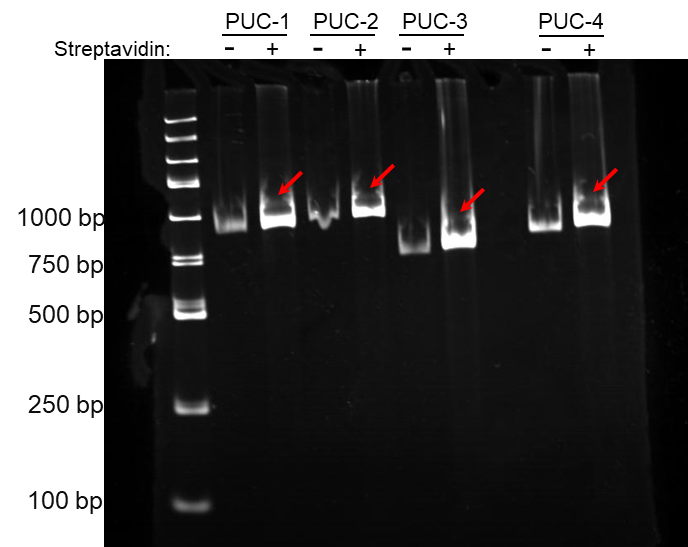


Figure S12. Representative gel for biotin-streptavidin-based strand shift of the εC lesion in the pUC-19 plasmid extracted from *E. coil* exposed to CAA. The shift bands are marked with red arrowheads.


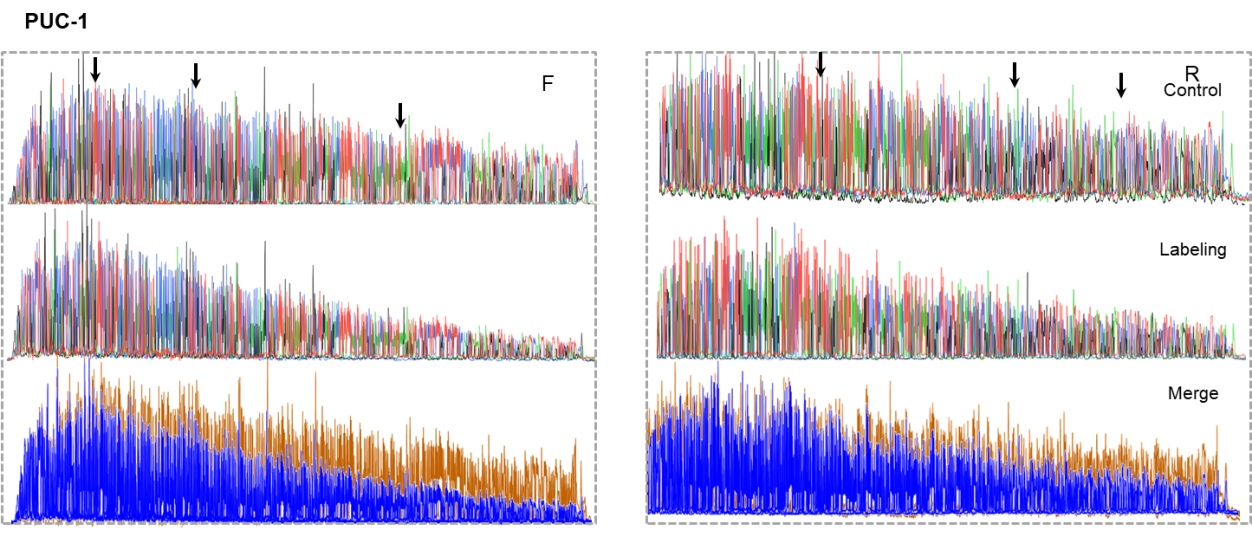


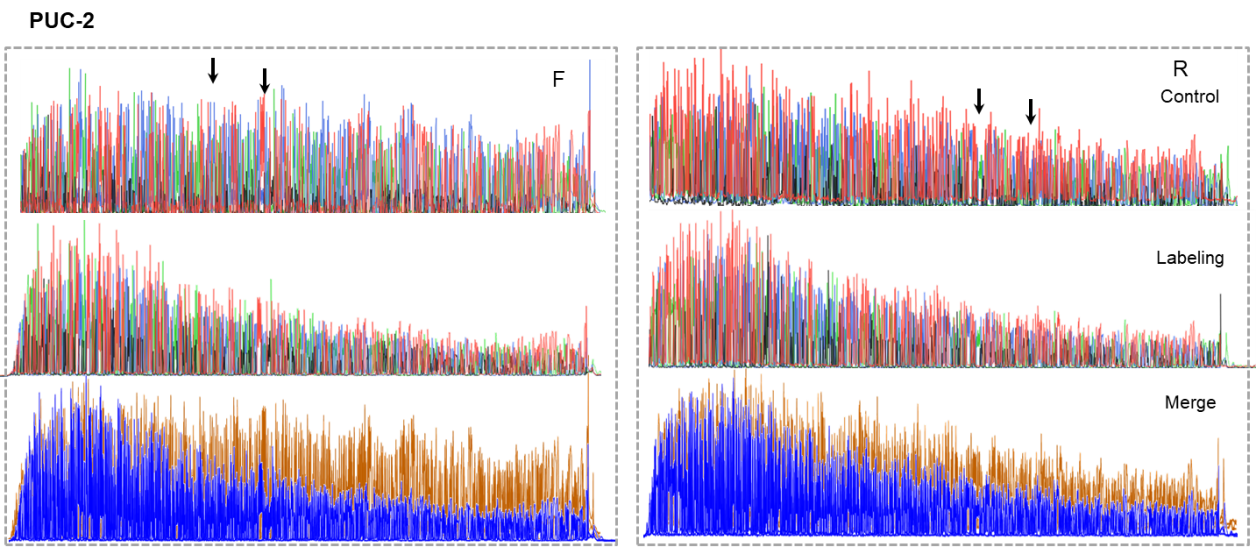


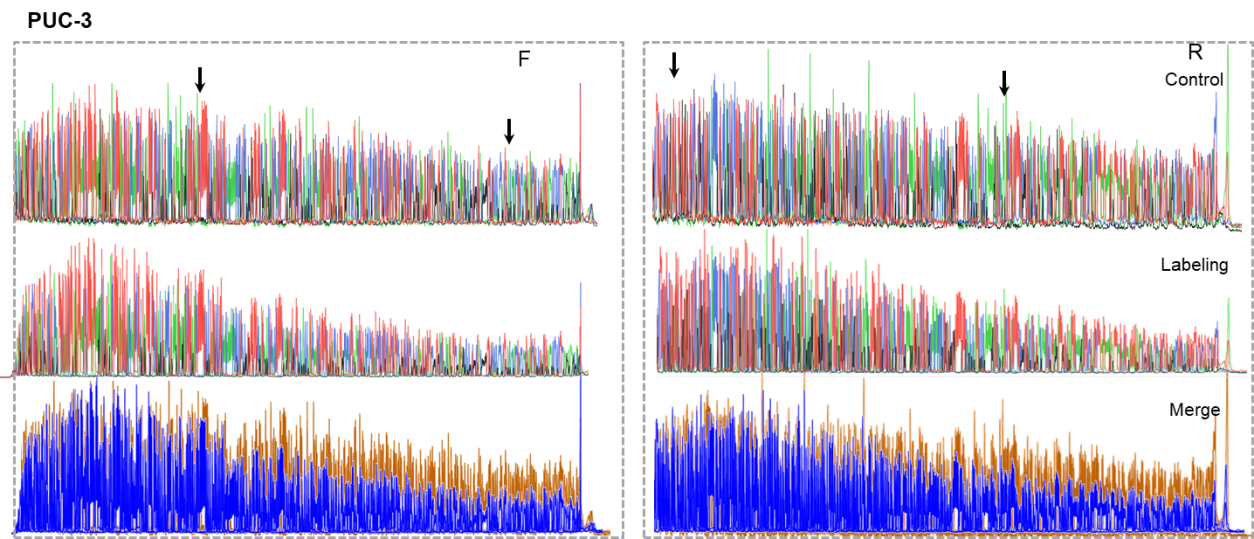


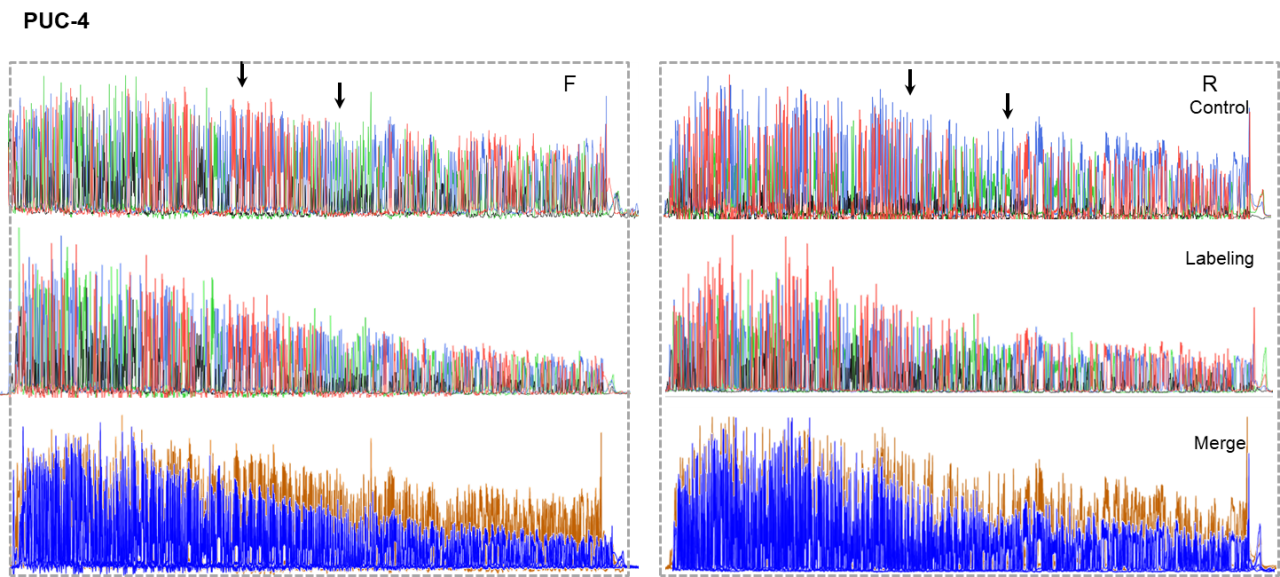


Figure S13. Sequencing of εC lesions labeled with TPT3-NaM pair in the pUC-19 plasmids extracted from *E. coli* exposed to CAA using primers PUC-1-4. Sense (F) and anti-sense (R) strands were both shown. Black arrowheads indicate regions with strong signal attenuation.


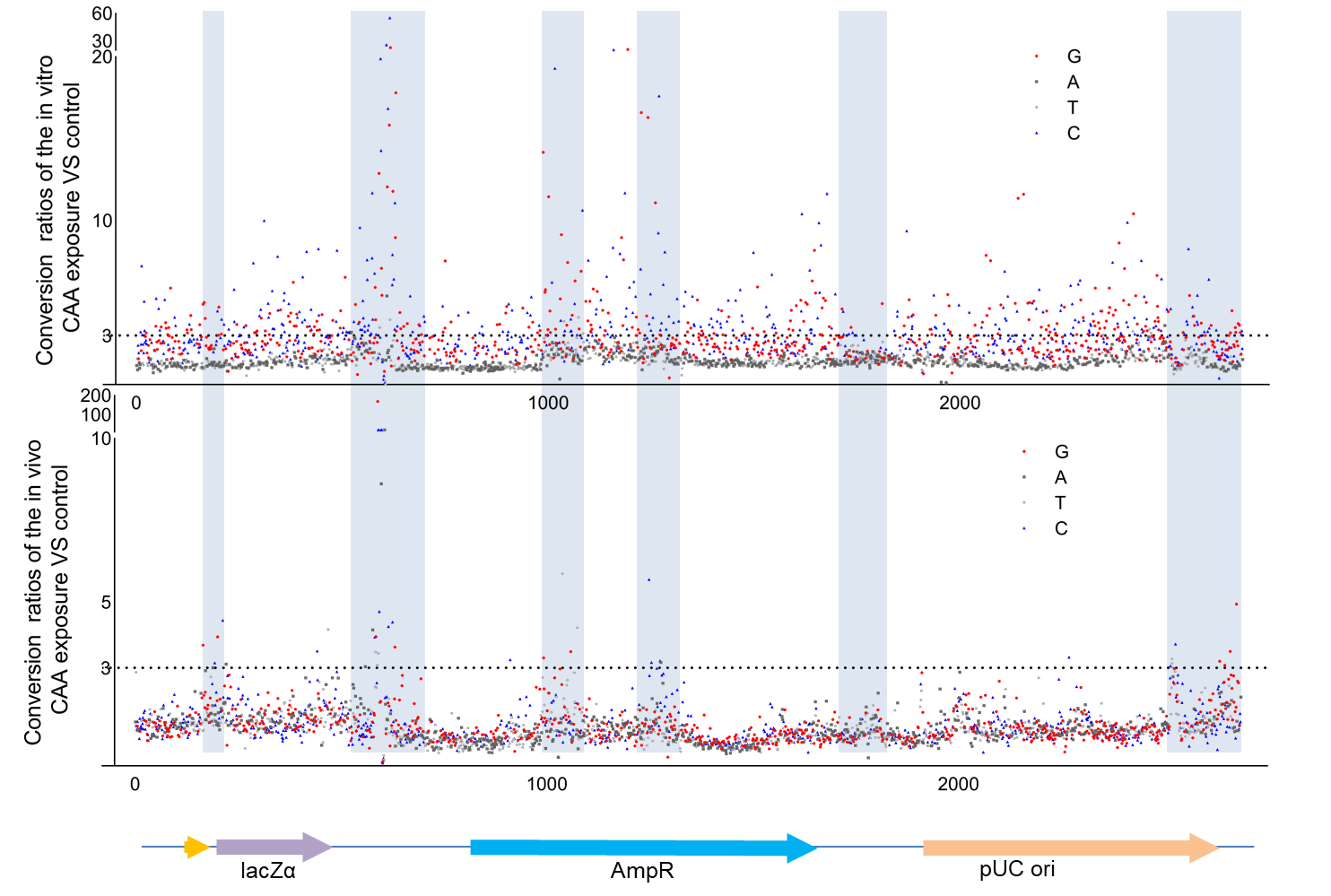


Figure S14. Comparison of the distribution of all the conversion ratios in the *in vivo* and *in vitro* damaged plasmids. Regions with similar conversion patterns are covered by blue.


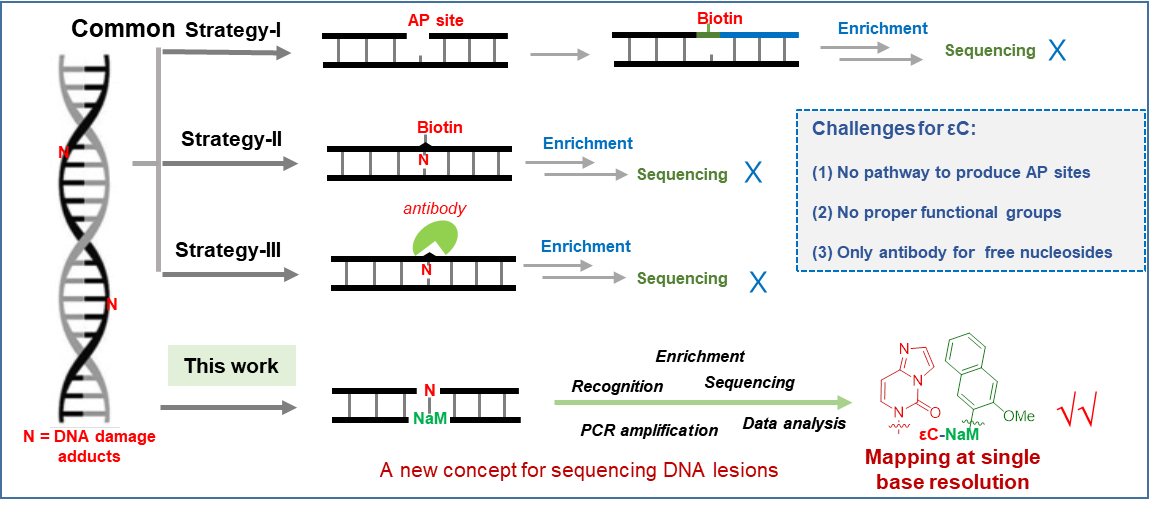


Figure S15. Strategies for sequencing DNA lesions.

Table S2. The distribution frequency of natural bases in each random site and the εC site after conversion and the same distribution from NaM-TPT3 of previous reported.

| **loci** | **T** | **A** | | **C** | **G** |
| --- | --- | --- | --- | --- | --- |
| -3 | 23.1±0.4/23.8±0.7 | 20.2±0.6/23.1±2.2 | | 28.2±0.7/27±1.8 | 28.5±0.2/26±1.7 |
| -2 | **23.9±0.3/25.1±0.4** | **20.8±0.5/25.3±1.9** | | **27.3±0.5/22.6±1.5** | **28±0.2/26.9±0.5** |
| -1 | **18.7±0.3/23.8±2** | 30.5±0.0/27.7±3.3 | | **31.8±0.2/21.9±1.1** | **19±0.1/26.5±0.5** |
| 0 | 3.9±0.6/5.3±1.5 | 4.3±0.4/14.4±6.3 | | 64.8±0.8/66.6±9.4 | 27±1.0/13.7±4.2 |
| 1 | **29.9±0.8/27.2±0.7** | **21.1±0.6/25.8±1.1** | | 22.6±0.8/22.9±0.4 | 26.4±1.0/24.1±0.7 |
| 2 | 23±0.7/25.8±1.8 | | **19.7±0.5/26.2±2.2** | **18.9±0.5/23.9±0.4** | **38.3±0.8/24.1±0.2** |
| 3 | 25.4±0.5/25.8±2.6 | | **12±0.3/25.3±2.5** | **27.5±0.8/25.5±0.6** | **35±0.1/23.3±0.1** |

Values are means ± SD (n=3), and bold values are statistically significant. “–” upstream, “+” downstream, values before “/” are sequenced in this paper, and values after “/” are sequenced previously as reported^[1]^.

# Reference

[1] H. Wang, W. Zhu, C. Wang, X. Li, L. Wang, B. Huo, H. Mei, A. Zhu, G. Zhang, L. Li, *Nucleic Acids Res* **2023**, *51*, e52-e52.
